# Life-Cycle Assessment and Costing of Fuels and Propulsion Systems in Future Fossil-Free Shipping

**DOI:** 10.1021/acs.est.2c03016

**Published:** 2022-08-23

**Authors:** Fayas Malik Kanchiralla, Selma Brynolf, Elin Malmgren, Julia Hansson, Maria Grahn

**Affiliations:** † Department of Mechanics and Maritime Sciences, Maritime Environmental Sciences, Chalmers University of Technology, SE-412 96 Gothenburg, Sweden; ‡ Sustainable Society, IVL Swedish Environmental Research Institute, Aschebergsgatan 44, SE-411 33 Göteborg, Sweden

**Keywords:** LCA, LCC, hydrogen, ammonia, methanol, battery, E-fuels

## Abstract

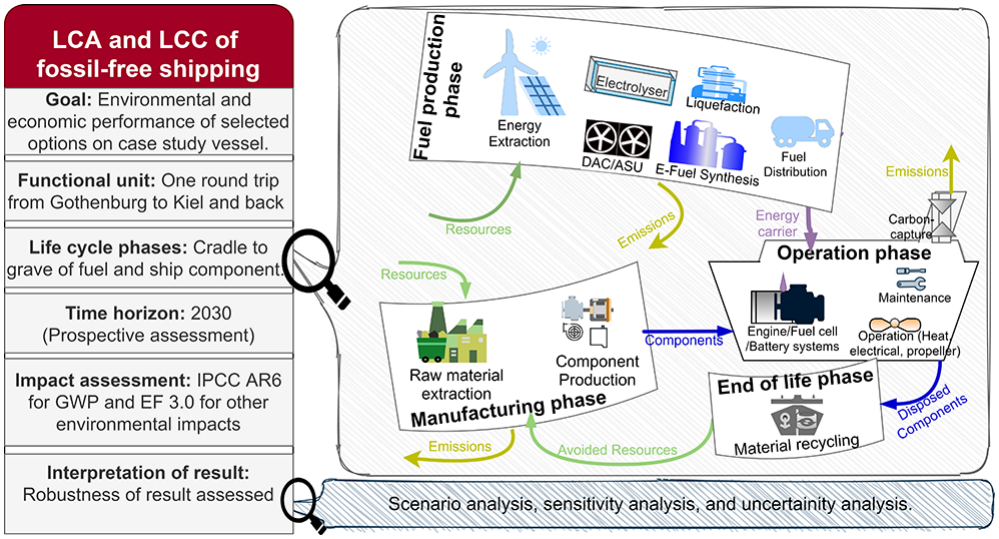

Future ships need to operate with low or possibly zero greenhouse gas (GHG) emissions while ensuring low influence on other environmental impacts and that the operation is economically feasible. This study conducts a life-cycle evaluation of potential decarbonization solutions involving selected energy carriers (electrolytic hydrogen, electro-ammonia, electro-methanol, and electricity) in different propulsion system setups (engines, fuel cells, and carbon capture technologies) in terms of environmental impact and costs. The results of the study show that the assessed decarbonization options are promising measures to reduce maritime GHG emissions with low-carbon-intensive electricity. The same order of GHG reduction is shown to be possible independent of the propulsion system and energy carrier used onboard. However, the carbon abatement cost ranges from 300 to 550 €/tCO_2_eq, and there is a trade-off with environmental impacts such as human toxicity (cancer and non-cancer effects) and freshwater ecotoxicity mainly linked with the wind infrastructure used for electricity production. Electro-ammonia in fuel cells is indicated to be effective in terms of the carbon abatement cost followed by the so-called HyMethShip concept. The higher abatement cost of all options compared to current options indicates that major incentives and policy measures are required to promote the introduction of alternative fuel and propulsion systems.

## Abstract 2

The paper presents the life cycle environmental and economic trade-offs for different electricity-based energy carriers in shipping and highlights the need for policies.

## 1 Introduction

The maritime sector is a central pillar of international trade and presently relies on fossil fuels like heavy fuel oil and marine gas oil (MGO).^1^ This sector is responsible for about 3% of the total global carbon dioxide (CO_2_) emissions contributing to climate change^2^ and other air emissions with a significant negative impact on air quality and human health.^1,3,4^ The International Maritime Organization (IMO) adopted a greenhouse gas (GHG) strategy in 2018 with the target to reduce the carbon intensity of shipping by at least 40% by 2030 and to reduce the total annual GHG emissions by at least 50% by 2050, compared to 2008.^5^ To reduce GHG emissions, energy efficiency measures, introduction of alternative fuels, and/or new propulsion technologies are required.^6−9^ Since the shipping trade is expected to grow and given the long service life of ships, to achieve the absolute reduction target of 2050, carbon intensity reduction of 75–85% per ton-mile would be required.^10^ Hence, by 2030, a significant proportion of new ships entering the market need to be prepared to adopt possible decarbonization solutions.^11^ Decarbonization may include the adoption of (i) low-climate-impact energy carriers (e.g., electrolytic hydrogen) and efficient energy conversion technologies (e.g., fuel cells), or (ii) onboard carbon capture (CC) systems (e.g., post-carbon capture).

Electricity,^12,13^ hydrogen (H_2_),^10,14^ ammonia (NH_3_),^10,14−19^ and methanol (MeOH)^10,14,20−24^ are potential low-climate-impact energy carriers when produced from sustainable biomass (called biofuels) or renewable electricity (often called electro-fuels, power-to-fuels, etc.) with different benefits and challenges. Onboard CC systems can decarbonize by capturing and storing CO_2_ in a ship that uses fuels containing carbon atoms. The CC technology may be post-combustion, pre-combustion, or oxyfuel combustion.^22,25−31^ The environmental performance of decarbonization solutions needs to be verified from a life cycle perspective as the impact may be shifted to different upstream and/or downstream activities.^32^ The inclusion of the manufacturing and end-of-life phases is however rare in previous life cycle studies for ship propulsion systems.^17,33^ The decarbonization solutions also need to be economically feasible in comparison to the present fossil-fuel-based system.

This study aims to investigate the different overall energy conversion, environmental performance, and economic conditions over the entire life cycle of eight decarbonization solutions using prospective life cycle assessment (pLCA) and environmental life cycle costing (eLCC). The following question is addressed: How do different life cycles’ use of energy and materials with related GHG emissions offsets the potential climate benefit of different decarbonization solutions, and are there other trade-offs in terms of energy requirement, environmental impact, and cost impact associated with the decarbonization options?

**Figure 1:**
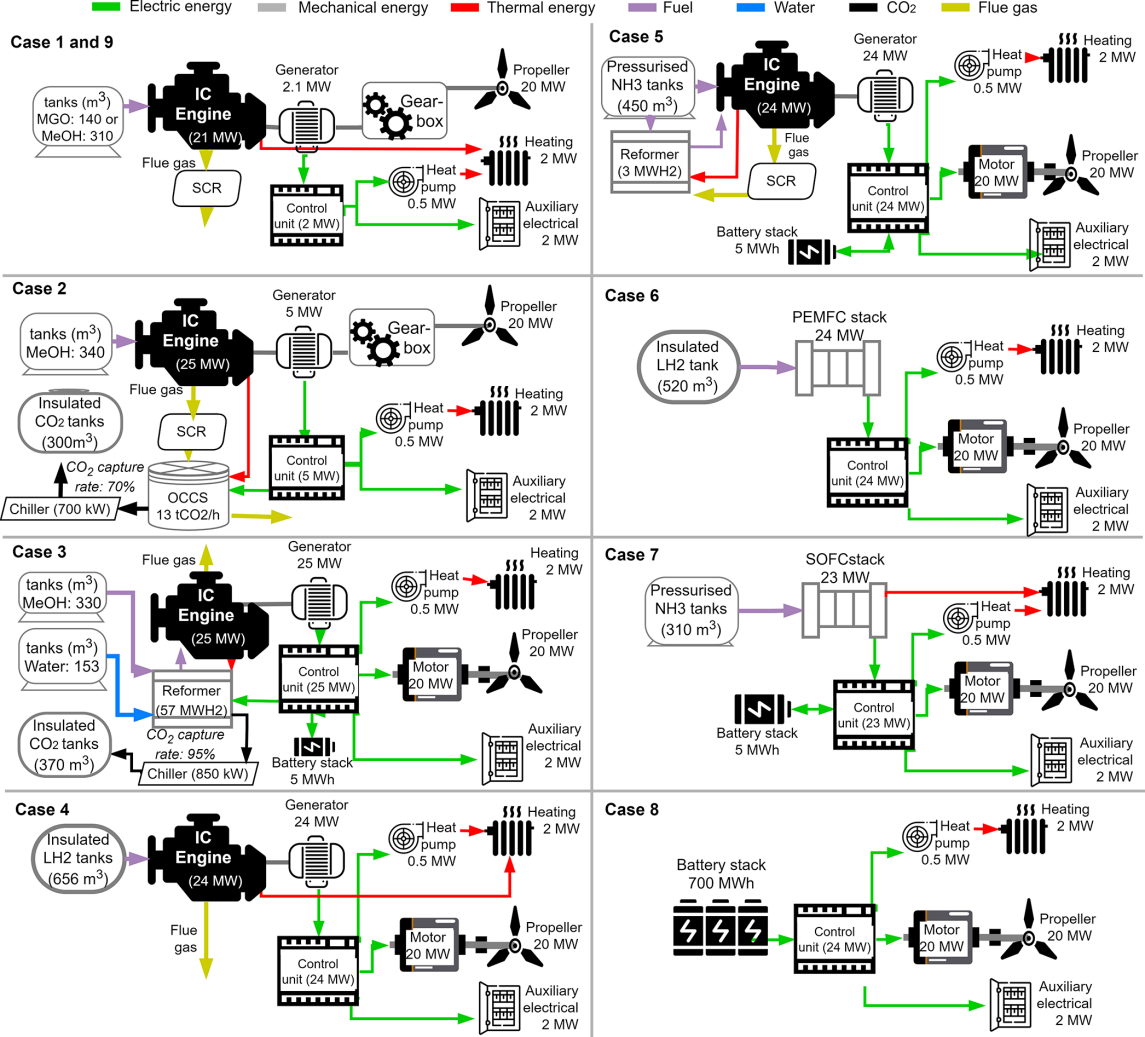
Propulsion system concept schemes considered in this study. Case 1 eMeOHICE: eMeOH in dual-fuel ICE with SCR and MGO as the pilot fuel; case 9 MGOICE: fossil MGO in medium-speed diesel ICE; case 2 eMeOHICE w PostCC: eMeOH in dual-fuel ICE with SCR and PostCC with a capture rate of CO_2_ from flue gases of 70%, with MGO as the pilot fuel, and a higher ICE power; case 3: the HyMethShip concept with a membrane reformer (pre-combustion CC) and separated H_2_ in the spark-ignition ICE and a CC rate of 95%; case 4 eLH2ICE: eLH_2_ in spark-ignition ICE; case 5 eNH3ICE: eNH_3_ in spark-ignition ICE with SCR where the pilot fuel is cracked H_2_ from the reformer; case 6 eLH2PEMFC: eLH_2_ in PEMFC with an electric motor; case 7 eNH3SOFC: eNH_3_ in SOFC (considered to have better compatibility with NH_3_^43^ than PEMFC) with an electric motor; and case 8 BE: batteries are sized for a round trip including a 30% reserve capacity and are assumed to be charged from the port of Gothenburg. An SCR is included for cases 1, 2, 5, and 9; the excess heat from the ICE/FC is used for heating load in cases 1, 4, 7, and 9. However, a heat pump is required during the mooring phase. Excess heat is not available for heating load in cases 2 (used for postCC operation), 3, and 5 (used for reformer operation). In cases 1, 2, and 9, the ICE powers the propeller directly and the shaft generator is used for meeting the electrical load.

The decarbonization solutions included are (1) electro-methanol (eMeOH) in an internal combustion engine (ICE), (2) eMeOH in ICE with PostCC, (3) the HyMethShip concept, which combines an onboard precombustion CC to separate H_2_ and CO_2_ with H_2_ ICE,^22^ (4) liquified electrolytic hydrogen (eLH_2_) in ICE, (5) electro-ammonia (eNH_3_) in ICE, (6) eLH_2_ in proton-exchange membrane fuel cells (PEMFCs), (7) eNH_3_ in solid oxide fuel cells (SOFCs), and (8) battery-electric (BE), as detailed in Figure 1. As seen, we have chosen to consider only one electro-fuel containing carbon (eMeOH). These solutions are emerging, and hence, the pLCA methodology is used where the emerging technologies are scaled to a reference technology level that is matured and has achieved a considerable level of market penetration.^34^ MGO in ICE (case 9) is the reference technology considered for comparison. Biomass-based decarbonization solutions are however not included as they have been extensively assessed previously and require a wider assessment in terms of sustainability and availability.^35,36^

The environmental performance of different shipping solutions using life cycle assessment has been investigated in several studies. Bilgili^17^ assessed ethanol, biodiesel, biogas, NH_3_, dimethyl ether (DME), and MeOH; Perčić et al.^18^ assessed BE, DME, H_2_, biodiesel, and MeOH; Hwang et al.^33^ assessed H_2_; and Malmgren et al.^22^ assessed MeOH and the HyMethShip concept. From a life cycle energy and cost perspective, Law et al.^37^ compared H_2_, NH_3_, MeOH, and BE. These studies have different system boundaries and assumptions, but all exclude the manufacturing and end-of-life phases of ship components. There are also studies assessing costs for ship propulsion systems, where, e.g., Korberg et al.^14^ and Stolz et al.^38^ have assessed the total cost of ownership for a range of different types of vessels and the attainment rate, respectively. For some results of these earlier studies, the reader is referred to the Discussion section. However, there is a lack of studies including entire life cycle assessment (LCA) and life cycle costs (LCC) with the same functional unit. This study is novel in the following three areas: (i) the pLCA includes the manufacturing phase of the components used in marine decarbonization solutions, (ii) it includes pLCA of both post- and pre-combustion onboard CC technologies including circular CO_2_ flow, and (iii) it includes eLCC of the decarbonization solutions.

## 2 Applied Methodology and Materials

The main steps of the pLCA and eLCC methodologies used in this study are based on standardized guidelines of ISO 14044:2006^39^ and are detailed in the Supporting Information (SI), Figure S1. The pLCA is used to investigate the expected environmental performance of the promising ship propulsion systems from the cradle to the grave (assuming a relatively mature stage). The eLCC is aligned with the pLCA in terms of scope definition, which includes the system boundaries, the functional unit, and methodological steps.^40^

The pLCA and eLCC methodologies are summarized in Table 1, and the study includes all shiploads of a case study Roll-On-Roll-Off-Passenger (RoPax) vessel further detailed in SI Section S1.1. The time horizon considered is 2030, and it is assumed that the vessel operates for 25 years.

**Table 1:** Summary of the pLCA and eLCC Methodologies

functional unit	one round trip from Gothenburg to Kiel and back with the case study ship	
time horizon	2030 (the time for which the ship propulsion systems are modeled and assumed more mature than at present)	
geographical boundaries	ship operation is limited to the North European ECA; component manufacturing, electricity generation, and fuel production are considered in Europe	
cost flows	expressed as annuitized cost in Euros (€) (with the base year 2021), considering the technical lifetime of the components and a discount rate of 3%	
life-cycle phases	• manufacturing phase (components)	• operation phase
	• fuel production phase	• end-of-life phase (components)
impact category^41^	• acidification	• human toxicity, cancer effects
	• climate change (GWP20 and GWP100)	• human toxicity, non-cancer effects
	• ecotoxicity freshwater	• ozone depletion
	• eutrophication marine	• particulate matter
	• eutrophication terrestrial	• photochemical ozone formation

### 2.1 Case Study Description

The eight decarbonization solutions and the reference case are shown in Figure 1 and described in the figure caption. Propulsion systems are modeled to comply with the Tier III NO*_x_* level (emission regulation set by IMO to reduce NO*_x_* emissions based on the rated speed of engines) as this is mandatory for new-built ships in North European Emission Control Areas (ECAs)^42^ and selective catalytic reduction (SCR) is considered for cases 1, 2, 5, and 9. A heat pump is considered for the heat requirement where excess heat is not available. In all cases, electricity from the respective port is used during the mooring phase. The components are sized and selected according to the shipload and vary based on additional loads and component efficiencies (detailed in the SI Section S1.3).

An electric motor is considered for propulsion in the cases with fuel cells (FCs) and BE (output is electricity) and spark-ignition ICE (for high torque requirement) based on expert opinion. For cases 1, 2, and 9, a direct drive transmission is considered. For cases 3, 5, and 7, a battery storage system is added to support startup and power ramping to operate the reformer. For eMeOH w PostCC and HyMethShip, the CO_2_ captured onboard is circulated back for eMeOH production. In all cases, the fuel storage systems are sized for round trips, including a 30% reserve capacity (also for the BE case). In total, five energy carriers are used in the decarbonization solutions: MGO, electricity, eLH_2_, eNH_3_, and eMeOH.

### 2.2 System Boundaries

**Figure 2:**
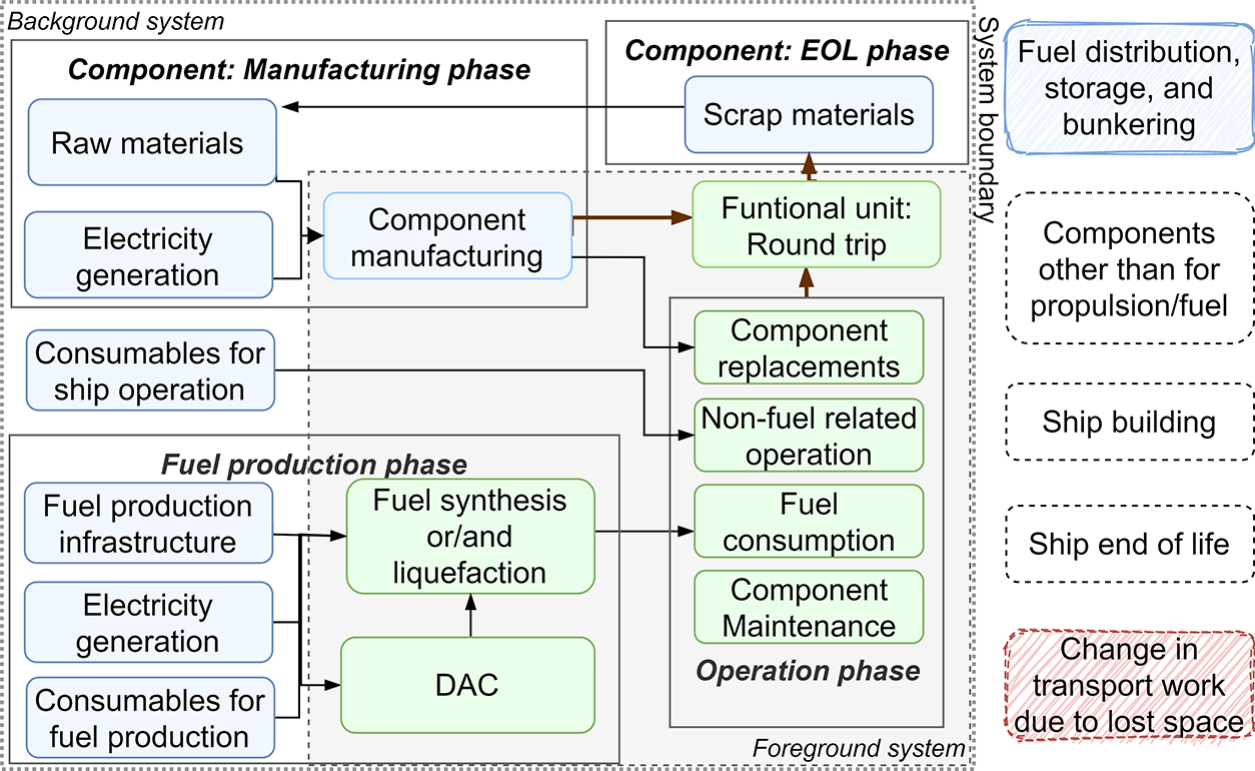
System boundaries including foreground and background systems. The foreground system includes processes that are focused on and modeled for the study, and all other processes are background processes. The processes inside the gray area are foreground processes in the pLCA. For eLCC, the green processes are the foreground processes, whereas the blue processes represent background processes. Processes that are not considered in the study are represented in white. However, the fuel distribution cost is considered in the scenario analysis marked in dashed blue. Transport work (marked in red) is not within the scope of the functional unit and hence not covered specifically in the LCA analysis. However, potential revenue loss due to lost space is included in the scenario analysis of the eLCC.

The processes in different life cycle phases are separated into foreground and background (Figure 2). For the pLCA, the foreground processes, in particular, are modeled at a future time^44^ and are scaled up to include technology development and use likely performance when relatively mature.^34,44^ The background processes are assumed to be static and include most of the upstream processes. Even though maintenance is associated with the components, the activity is performed during the operation phase.

### 2.3 Environmental Inventory Analysis and Assessment

For the foreground processes, the input and output material flow, energy flow, and emissions are collected from different sources, including scientific articles, reports, catalogs, lab experiments, and results from pilot projects. In addition, 10 sets of interviews were conducted with various experts from relevant fields for their opinion using a structured set of questions. The questions include the inventory collection over life cycle phases in different time horizons based on their opinion on the likely development of the novel technologies. For the background processes, the LCI data are taken from Ecoinvent v3.7.1.^45^ By choosing a background process, temporal mismatch with the foreground system should be avoided.^44^ Temporal changes in the electricity mix of the grids are adjusted to the scenario projection for the year 2030 based on the European Commission 2020 reference scenario.^46^

#### 2.3.1 Fuel Production Pathways

MGO from an average EU petroleum refinery including all upstream processes (Ecoinvent 3.7.1)^45^ is used. The electricity used in all fuel production processes is from onshore wind (Ecoinvent 3.7.1), and a capacity factor of 41% is considered.^45^ The electrolytic-H_2_ (eH_2_) is produced from deionized water^45^ and electricity using an alkaline electrolyzer.^47^ eH_2_ is used as feedstock to produce eNH_3_ and eMeOH. For direct use (cases 4 and 6), H_2_ liquefaction^48^ using electricity is considered to increase the volumetric energy density. eH_2_, captured CO_2_, and electricity are used for eMeOH synthesis.^49^ For case 1, all CO_2_ needed for eMeOH synthesis is considered from direct air capture (DAC),^50^ but for cases 2 and 3, the captured CO_2_ from the ship is used and complemented with DAC (31% for case 2 and 6% for case 3 come from DAC considering resupply from the ship and CO_2_ leakage in resupply). Electricity, eH_2_, and captured nitrogen are used for eNH_3_ synthesis using the Haber–Bosch process.^51^ Nitrogen is considered from the cryogenic air separation unit (ASU).^52^Table 2 shows the important operation and cost parameters for the different processes in each production pathway, and more details are provided in the SI Section S2.1. We have made the simplified assumption that the rate of CO_2_ capture exactly matches the demand for CO_2_ in electro-fuel production, as well as that the rate of fuel production exactly matches the demand for fuels, implying that there is no need for storing carbon or fuels. We, further, disregard that there may be a need (and thus a cost) for carbon and fuel transport. Moreover, the potential need for electricity storage due to the variability of renewable energy sources is uncertain and not considered specifically in this study. For the BE option (case 8), the batteries are assumed to be charged from the Swedish grid electricity mix at port 1 (Gothenburg). Battery charging using renewable energy during the limited time available in the port requires energy storage at the port.

**Table 2:** Technical and Cost Parameters for the Fuel Production Pathways Considered^a^

	operation parameter		cost parameters		infrastructure	
	main parameter	lifetime (years)	CAPEX	O&M cost^c^ (% of CAPEX/year)	ref	ref
onshore wind	41%^b^	30	1.04 M€/MW	4%	(54)	(45)
electrolysis	50 kWh/kgH_2_	30	450 €/kW	5%	(55, 56)	(55)
NH_3_ synthesis	0.472 kWh/kgNH_3_	30	174 k€/tNH_3_/day	5%	(51, 52, 56)	(45)
MeOH synthesis	0.858 kWh/kgMeOH	30	69 k€/tMeOH/day	5%	(49, 56, 57)	(45)
H_2_ liquefaction	6.4 kWh/kgH_2_	25	2100 €/kgLH_2_/day	4%	(58)	(58)
ASU	0.314 kWh/kgN_2_	30	376 €/kgN_2_/day	5%	(52)	(45)
DAC	0.875 kWh/kgCO_2_	30	271 €/kgCO_2_/day	5%	(50, 59)	(50)

a The data for the fuel production infrastructures are adopted from the references mentioned in the last column. MGO price is assumed as 600 €/tonne based on the average price of 2021.^53^

b Capacity factor.

c Including fixed O&M cost but does not include consumable cost and electricity cost.

#### 2.3.2 Component Manufacturing and EOL

The components’ operation and cost parameters are shown in Table 3. Only raw material flow and electricity (EU mix) for manufacturing of the components are considered, and LCI data are analyzed based on the literature reviews and expert opinions (details given in the SI Section S2.3). A cutoff approach is used for including EOL of materials used in manufacturing, where a share of secondary raw material is assumed for manufacturing following the Ecoinvent database.

**Table 3:** Major Technical and Cost Parameters of the Propulsion System Components Used in the Study^a^

component	major parameter	lifetime (years)	specific CAPEX cost	O&M cost (% of CAPEX/year)	refs	material data
MS ICE, diesel	48 kW_Mech_/KWh_fuel_	25	240 €/kW	2%	b(14),	(60, 61)
DF ICE, MeOH	48 kW_Mech_/KWh_fuel_	25	265 €/kW	2%	b	(60, 61)
SI ICE, HyMeth	42 kW_Mech_/KWh_fuel_	25	350 €/kW	2%	b	(60, 61)
SI ICE, H_2_	44 kW_Mech_/KWh_fuel_	25	350 €/kW	2%	b	(60, 61)
SI ICE, NH_3_	44 kW_Mech_/KWh_fuel_	25	350 €/kW	2%	b	(60, 61)
PEMFC	55 kW_el_/KWh_fuel_	8	1100 €/kW	2%	b(62),	(63)
SOFC	60 kW_el_/KWh_fuel_	8	2500 €/kW	2%	b	(64)
electric motor	98% efficiency	25	120 €/kW	1%	(65, 66)	(45)
gearbox	98% efficiency	25	85 €/kW	1%	(14, 65)	(61)
MeOH reformer	0.05 kW_th_/kW_H2_	25	475 €/kW_H2_	2%	b	b
NH_3_ reformer	0.05 kW_th_/kW_H2_	25	475 €/kW_H2_	2%	b	b
alternator	97% efficiency	25	120 €/kW	1%	(66, 67)	(68)
SCR system	NA	13	40 €/kW	2%	(16, 69)	(70)
CO_2_ chiller	0.0645 kWh/kgCO_2_	25	102 €/kgCO_2_/h	2%	(71, 72)	(45),^b^
battery	89% efficiency	8	200 €/kWh	1%	(73, 74)	(75)
Heat pump	4 COP^c^	25	1000 €/kW	2%	(76, 77)	(78)
postCC	98.3 Wh_el_/kgCO_2in_	25	3500 €/kgCO_2_/h	3%	(72, 79)	(80)
tank, MGO	NA	25	0.09 €/kWh	2%	(14)	b
tank, MeOH	NA	25	0.14 €/kWh	2%	(14)	b
tank, NH_3_	0.1% daily BOG^d^	25	0.29 €/kWh	2%	(14, 20)	b
tank, LH_2_	1.5% daily BOG^d^	25	1.71 €/kWh	2%	(20)	b
tank, CO_2_	1% daily BOG^d^	25	0.6 €/kg	2%	(14)	b

a The raw material for each component below is detailed in the SI, and relevant references used are shown in the last column. O&M cost includes only fixed costs and does not include fuel and consumable costs.

b Based on expert interviews.

c Coefficient of performance.

d Boil off-gas; Mech, mechanical output; el, electrical output; th, thermal input.

#### 2.3.3 Operation Phase

The main input flows are energy, consumables, and the replacement of components (if required), and output flows are mainly shiploads and emissions due to fuel combustion, which depend on the emission factors of ICE/FC technology. The amount of fuel consumed depends on the efficiencies of the components, shipload, additional load, and excess heat available, as listed in Table 4. Another important flow in the operation phase is consumables for the operation (e.g., PostCC, SCR). Since the fuel consumption and emissions vary with engine load, in this study, two loads (80% during cruising and 20% during maneuvering) are considered (Table 4). The emissions from engines during operation are mostly released as exhaust gases.^22^ Emissions to water and soil are mainly related to bilge water and stern tube oil^81^ and are not considered in the study since it is assumed that these emissions would be similar for all cases. FC options are assumed to have cleaner electrochemical oxidation.^82^ One of the limitations of this study is that it excludes the changes in operation pattern due to the volume and weight changes due to the addition of components like PostCC, reformer, batteries, cryogenic storage tanks, other electrical systems, and modifications required for ICEs and FCs. These additional changes are complex as depending on where the additional weight is placed, there would be a modification in the placement of ballast water and other components to optimize the center of gravity.

**Table 4:** Inventory Data of Emissions from the Combustion of Fuel in Different ICE Technologies^a^

fuel/option	MGO^2,83,84^		methanol^2,83−85^		HyMethShip^22^		hydrogen^22^		ammonia^22,83^	
ICE type	MS, CI		DF MS ICE		SI ICE		SI ICE		SI ICE	
TRL level	9		8		5		5		3	
ICE load	80%	20%	80%	20%	80%	20%	80%	20%	80%	20%
SFC (g/kWh)	175	202	370	428	75	70	68	73	435	467
NH_3_ (g/kWh)	0.04	0.04	0.01	0.01					0.04	0.04
BC (g/kWh)	0.026	0.147	0.011	0.013						
CO_2_ (g/kWh)	561	647	508	588						
CO (g/kWh)	1.10	2.20	6.60	3.70	0.129	0.004	0.129	0.004	0.129	0.004
N_2_O (g/kWh)	0.013	0.013	0.003	0.003					0.013	0.013
CH4 (g/kWh)	0.01	0.01	0.02	0.04						
NO*_x_* (g/kWh)	2.60	2.60	2.60	2.60	0.784	1.589	0.784	1.589	2.60	2.60
NMVOC (g/kWh)	0.527	0.527	0.053	0.053	0.003	0.0	0.003	0.0	0.003	0.0
PM_10_ (g/kWh)	0.180	0.180	0.140	0.140	0.021	0.013	0.021	0.013	0.021	0.013
PM_2.5_ (g/kWh)	0.166	0.166	0.129	0.129						
SO*_x_* (g/kWh)	0.245	0.283	0.05	0.074						
urea req. (g/kWh)	7.1	7.1	2.6	6.4						
pilot fuel			MGO	MGO					H_2_	H_2_
SFC of pilot fuel			2	4					3.57	3.85

a The ICE load of 80% for cruising and 20% of ICE load for maneuvering are assumed. Emissions not listed are assumed zero.

#### 2.3.4 Environmental Impact Assessment

Ten impact categories are analyzed (Table 1). For life cycle impact assessment (LCIA), a midpoint-level approach is used where global warming potential GWP20 and GWP100 impact categories are based on the sixth assessment report (AR6) of the Intergovernmental Panel on Climate Change (IPCC),^86^ and other impact categories are assessed according to the Environmental Footprint (EF) 3.0 method.^41^

The total environmental impact results (IRs) for different categories (C) are calculated to the functional unit from the characterization factor (CF) of the substance (*i*) as in the respective LCIA method and the amount of substance (*m*_*i*_) emitted to the environment using eq 1.
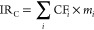
1

#### 2.3.5 Normalization

Normalization is optional as per ISO 14044^39^ but provides a reference situation for the environmental pressures^87^ as environmental impact interpretation is difficult to understand without a reference.^32^ In this analysis, the global normalization factors (NFs), representing the relevance of the total environmental impact in a certain category in a global context, are taken from EF 3.0.^88^ The normalized value (NV) is calculated using eq 2, where *c* represents the impact category.
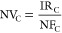
2

### 2.4 Economic Inventory Analysis and Assessment

The same methodology as for data collection is used for cost flows. The eLCC includes all upstream cost flows in the background system.^40^ In this study, the manufacturing and EOL phase is considered in the background system, and the final cost of purchasing and the scrap value are calculated separately. The eLCC including all of the costs for the round trip is shown in eq 3, where *C*_A_ is the acquisition cost, *C*_F_ is the fuel cost, *C*_C_ is the cost of consumables, *C*_M_ is the maintenance cost, *C*_R_ is the replacement cost*, C*_O_ is the operation/overhead cost, *C*_E_ is the external cost, and *C*_EOL_ is the disposal cost. The detailed calculation methodology is given in the SI Section S1.4.
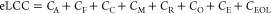
3

The carbon emission abatement cost (defined by eq 4 and using GWP100) is used to compare the technology options considering both GHG emissions and costs.^89^
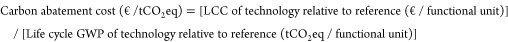
4

### 2.5 Interpretation of Results

The reliability and robustness of the results are studied. Many of the technologies in focus in this study are in their early stages of development. The possibility of altering and controlling is therefore high, and only limited knowledge is presently available, meaning that assumptions on future technologies largely depend on technology development.^44^ Three approaches are used to assess the robustness of the results, i.e., sensitivity analysis, scenario analysis, and uncertainty analysis including different parameters (detailed in Table 5).^39^ The LCA results do not cover the effect of volume and weight changes due to the shipping solutions on the transport work (e.g., tonne-km or person-km), which is due to the functional unit used. However, for the cost analysis, these effects are assessed, to some extent, in the scenario analysis by assessing the impact of revenue loss.

**Table 5:** Robustness of Results Is Analyzed Using Sensitivity Analysis, Scenario Analysis, and Uncertainty Analysis on the Parameters that may Affect the Results the Most. (Min, Minimum Value; Max, Maximum Value)^a^

description of parameter	parameter ranges or scenario
Sensitivity Analysis	
carbon dioxide intensity for different electricity mixes for energy use	the carbon footprint of the electricity supply is varied from 0 to 300 kgCO_2_eq/kWh.
cost effect of different carbon allowance scenarios on the eLCC	the impact of a carbon tax from 50 to 400 €/tCO_2_ (for fossil-based CO_2_ emissions from fuel use) is analyzed.
Scenario Analysis	
battery options based on charging frequency and battery swapping options	*base (case 8 or 8a)*: Onboard batteries for a round trip charged only using Swedish grid alone (base case)
	*case 8b*: Onboard batteries only for a single trip charged at the Swedish grid (50%) and the German grid (50%)
	*case 8c*: Onboard batteries for a single trip, additional sets of batteries at both ports, which enable charging from wind power based on wind availability
fuel distribution and storage costs	a case considering the cost for fuel distribution is included (d*etails in Table S13*)
revenue loss	income loss is associated with the additional volume required to accommodate fuel and components; the assumed rate is 8 €/m^3^ ^14^
Uncertainty Analysis (Monte Carlo)	
CO_2_ capture rates for PostCC and precombustion carbon capture	*post-carbon capture rate*: min: 60%; base case: 70%; max: 90%
	*pre-carbon capture rate*: min: 90%; base case: 95%; max: 98%
batteries and FCs have less operational life compared to the lifetime of the ship	*number of replacements*: min: zero; base case: 2; max: 2
daily leakages of liquefied fuel during distribution and bunkering^20^	*LH*_*2*_: min: 0.75%; base case: 1.5%; max: 3%
	*NH*_*3*_: min: 0.05%; base case: 0.1%; max: 0.2%
efficiency of ICE/FCs and battery energy storage capacity	*SI Otto ICE (cases 4, 5)*: min: 42%; max: 46%
	*HyMethShip ICE (case 3)*: min: 41%; max: 45%
	*CI diesel ICE (cases 1, 2, and 9)*: min: 46%; max: 50%
	*PEMFC (case 6)*: min: 52%; max: 57%
	*SOFC (case 7)*: min: 58%; max: 62%
	*battery capacity (Wh/kg) (case 8)*: min: 180; max: 240
N_2_O emission from NH_3_ ICE	*emission varied from 0.013 to 0.13 g/kWh*
energy use for the processes in fuel production	*electrolysis (kWh/kg*_*H2*_*)*: min: 47; max: 53
	*H*_*2*_*liquefaction (kWh/kg*_*H2*_*)*: min: 6; max: 7
	*NH*_*3*_*synthesis (kWh/kg*_*NH3*_*)*: min: 0.333; max: 0.874
	*eMeOH synthesis (kWh/kgMeOH)*: min: 0.437; max: 1.292
	*DAC (kWh/kgCO*_*2*_*)*: min: 0.600; max: 1.230
cost effect of the efficiencies and infrastructure cost on fuel cost and eLCC	*electrolysis (€/kW)*: min: *350;* max: *570*
	*NH*_*3*_*synthesis (k€/tNH*_*3*_*pd)*: min: *160*; max: *215*
	*eMeOH synthesis (k€/tMeOHpd)*: min: *46;* max: *46*
	*electricity cost (€/MWh)*: min: *30;* max: *70*
	*battery cost (€/kWh)*: *min*: *180;* base case: *200;* max: *220*

a The uncertainty analysis was performed using Monte Carlo simulation with uniform distribution of the range of parameters with 10,000 iterations.

Leveling of intermittent renewable energy is assumed to be done within the grid (i.e., excess electricity produced is sold to the grid, and vice versa). There may also be additional storage requirements for H_2_, CO_2_, N_2_, and respective fuels to ensure continuous operation, which has not been evaluated in this study mainly due to the uncertainty. The connection between electricity prices and demand for different types of energy storage is complex and differs between regions and depends, for example, on the flexibility in fuel production. An analysis of how this connection affects the production costs of electro-fuels can be found in.^90^ In this study, the electricity price is varied using uniform distribution in the Monte Carlo simulation along with other production costs.

## 3 Results

### 3.1 Energy Conversion Efficiency

**Figure 3:**
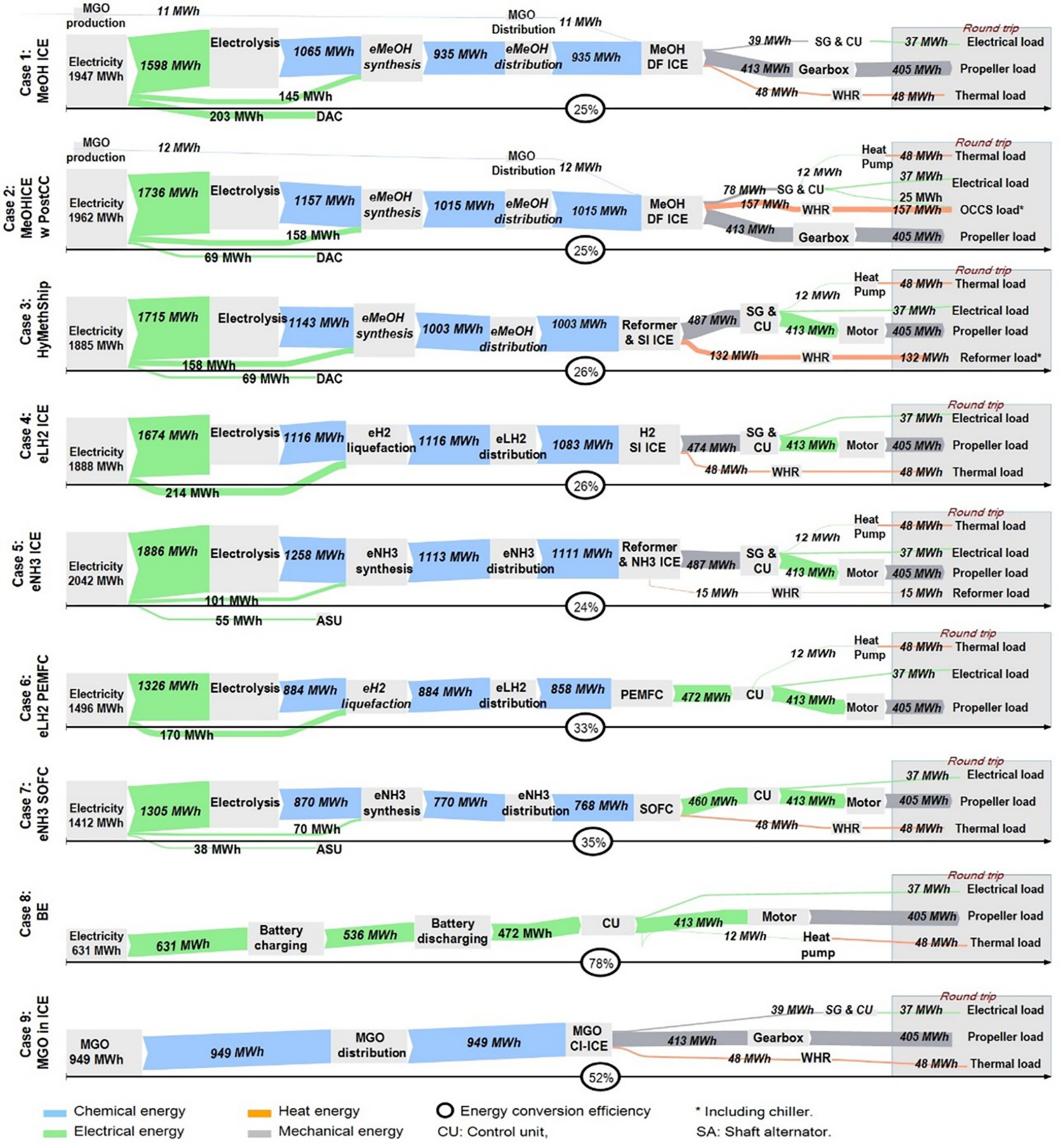
Energy conversion efficiency for the major conversion processes from pathways starting from the base energy carrier, i.e., electricity or MGO, to useful work for different cases compared. The conversion losses from primary energy to MGO or electricity are not included in the study.

The conversion efficiencies of the studied decarbonization options from the use of electricity and MGO to the final use of the energy carriers are shown in Figure 3. The fuels eLH_2_, eNH_3_, and eMeOH are produced from electricity and linked with additional conversion losses during both the upstream (production) and downstream (conversion) steps compared with MGO and BE. Among all options, eNH_3_ and eMeOH pathways have the lowest round-trip energy conversion efficiency due to higher losses in upstream processes. For the HyMethShip concept, efficiency is slightly higher than PostCC systems. More heat is available when operating PostCC (see Figure 3); this is because a lower temperature is required for PostCC (120 and 160°C)^91^ than in a MeOH and NH_3_ reformer (above 200 and 350 °C, respectively).^92^ Compared to MGO, the investigated fuels used in ICE require 2–2.5 times more energy, whereas their use in FCs requires around 1.5 times more energy. BE requires 40% less energy than MGO (it may be noted that the chemical energy in MGO is compared with electrical energy). The relative comparison of energy conversion efficiencies used intermediate energy carriers (electricity, fossil product) rather than primary energy. This intermediate product can either be directly used on ships (e.g., MGO or electricity in batteries) or be converted to other energy carriers used on ships. The different cases are not assessed from an exergy perspective, i.e., considering different qualities of different energy carriers, but this could be explored in future assessments.

### 3.2 Environmental Impact Assessment

#### 3.2.1 Global Warming Potential

**Figure 4:**
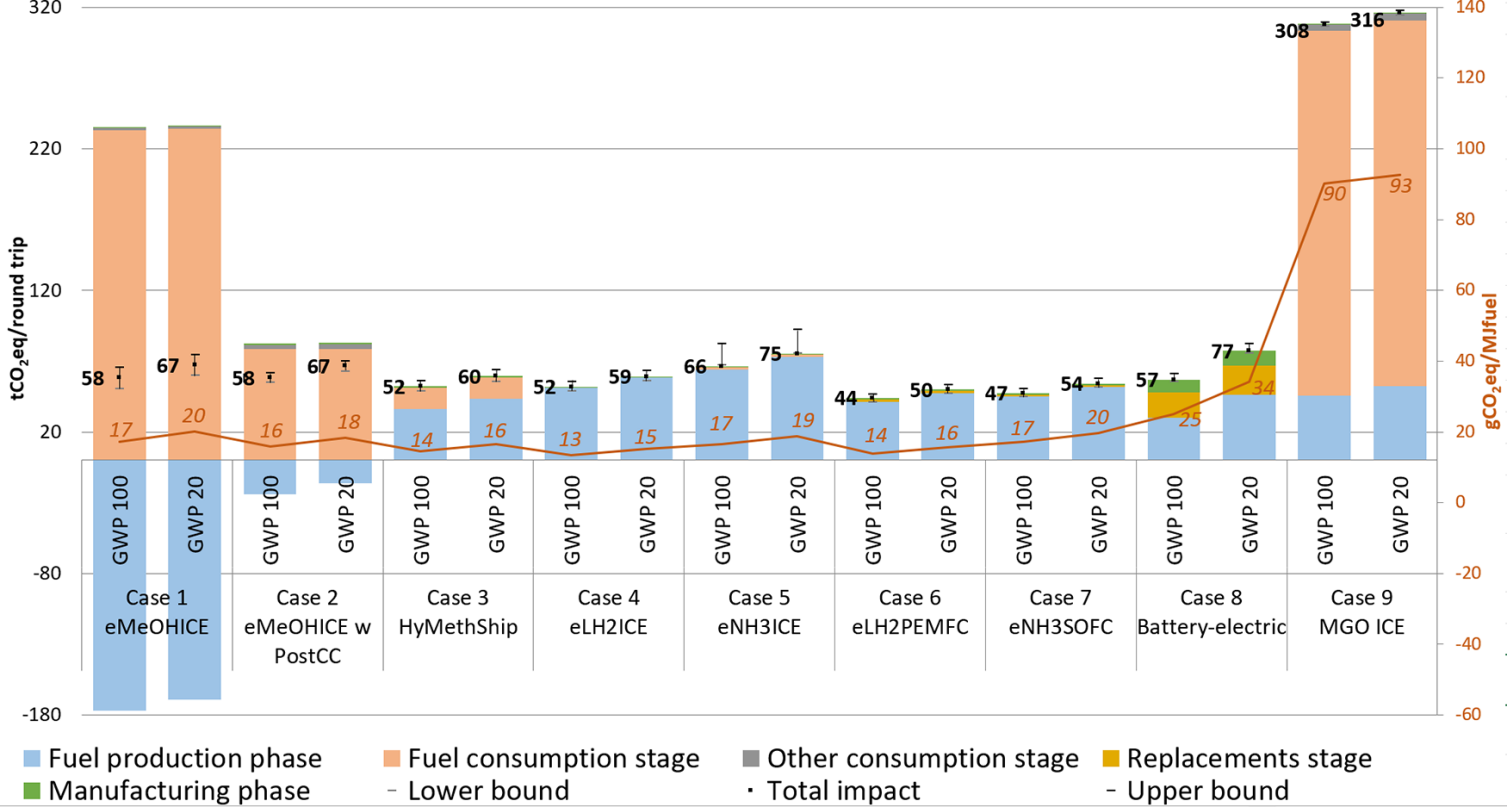
pLCA results on climate change potential (GWP20 and GWP100) for the round trip. The results are divided into five parts, including fuel production, fuel consumption, other consumptions, replacement, and manufacturing. For cases 1 and 2, the negative impact of fuel production is because the CO_2_ for eMeOH synthesis is captured from air and for case 2 due to the fact that not all CO_2_ is captured. For case 3, the majority of CO_2_ for eMeOH synthesis comes from recirculation. Arrows indicate that the secondary axis is applicable for gCO_2_eq/MJfuel, which represents the GWP impact per fuel required for the respective options. Uncertainty range from the Monte Carlo simulation, where the upper bound representing the 95 percentile and the lower bound representing the 5 percentile are also included.

The pLCA results on GWP20 and GWP100 for the studied cases are shown in Figure 4. In general, all of the pathways could reduce climate change significantly, with the highest reduction potential for eLH2PEMFC followed by eNH3SOFC and eLH2ICE. For the carbon-based energy carriers, the fuel combustion stage contributes most to climate impact. For carbon-free energy carriers, the fuel production phase and mainly electricity generation contribute most to climate change. The influence of assuming only renewable electricity production for the electricity used in batteries for BE is analyzed in the scenario analysis in the SI Section 3.2. Manufacturing of the components and their replacements have a relatively low climate impact, with the highest being for battery production. The result of the Monte Carlo simulation is shown as the uncertainty bar in Figure 4. The relatively large uncertainty for GHG emissions for eNH3ICE (+25%) is mainly due to the uncertainty associated with the N_2_O emissions (having a characterization factor of 273). The uncertainties for the other options are around ±8%, showing that the GWP reduction potential of these options remains very high (given that the fuel is produced from wind power).

#### 3.2.2 Other Environmental Impact Categories

**Figure 5:**
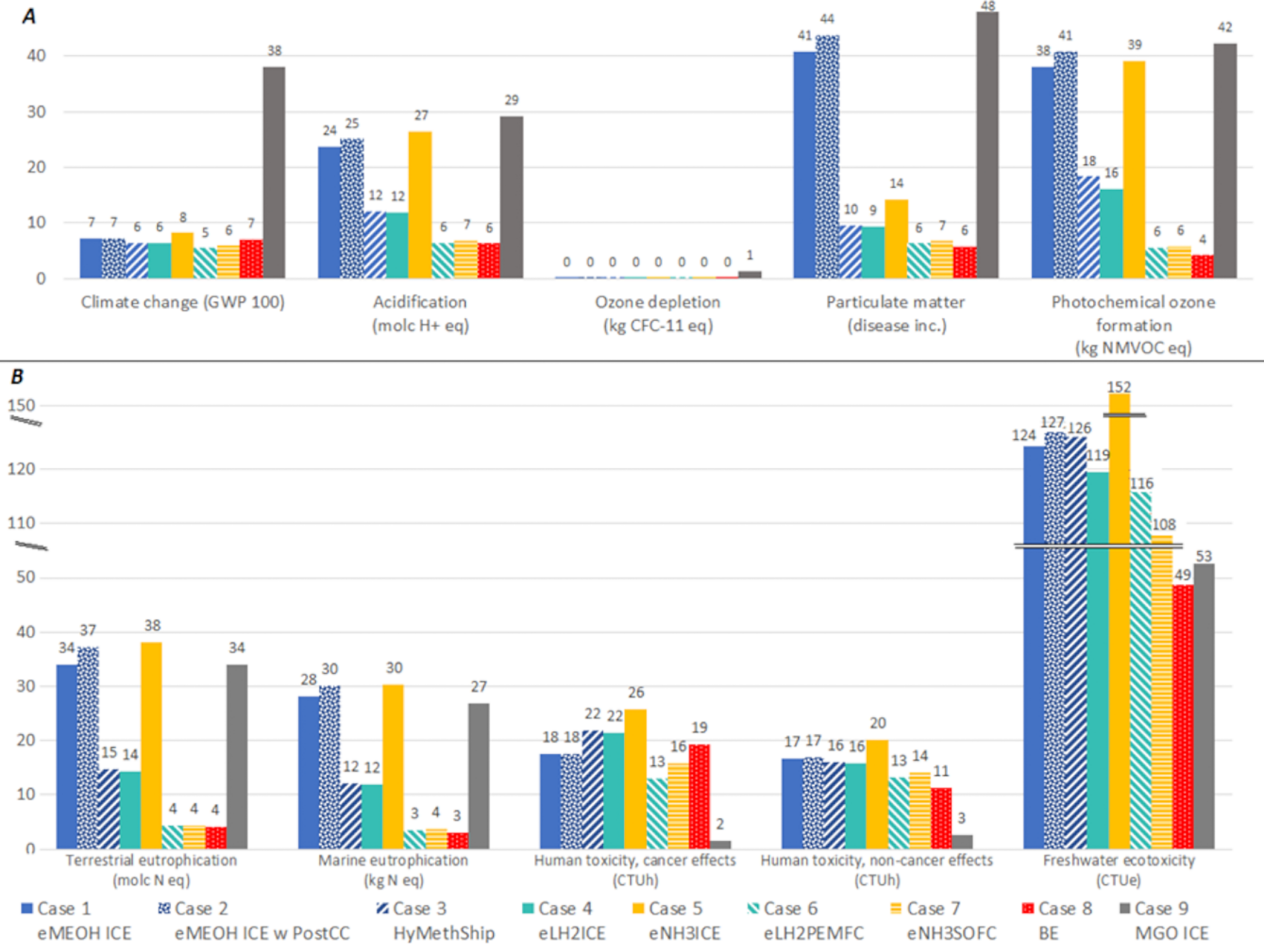
Normalized results based on EF 3.0 from pLCA for different impact categories. (A) Environmental impacts where all decarbonization options have a lower impact. (B) Impact categories that have different impacts compared to the reference case operating on MGO.

Figure 5 shows the normalized results of other environmental impacts (detailed results before normalization are shown in SI S3.1). All decarbonization solutions have lower acidification, ozone depletion, photochemical oxidation, and particulate matter impacts than the reference case. Acidification is foremost affected by SO_*x*_ and NO*_x_* emissions, and around 70% of the impact for eMeOHICE (case 1), eMeOHICE w PostCC (case 2), and eNH3ICE (case 5) is linked with NO*_x_* emissions and NH_3_ slip from the ICE after SCR. The remaining impact is from the fuel production phase. For the HyMethShip (case 3), eLH2ICE (case 4), eLH2PEMFC (case 6), eNH3SOFC (case 7), and BE (case 8), the acidification impact is mainly associated with fuel production. In the fuel production phase, the major contributor is the wind power infrastructure (materials like copper, chromium steel, aluminum, etc.). Photochemical oxidation and particulate matter impacts also have a similar result pattern.

For marine eutrophication and terrestrial eutrophication (Figure 5B), cases 1, 2, and 5, i.e., eMeOHICE, eMeOHICE w PostCC, and eNH3ICE, have a higher impact than the MGOICE. Even though the major effect (around 85%) of eutrophication is associated with emissions from ICEs similar to MGO, the added impact from the electricity production results in that these options have a higher impact than the MGO case. The human toxicity (cancer effects and non-cancer effects) and freshwater ecotoxicity impacts are higher for the studied options compared to MGO and are mainly related to the wind power infrastructure used for electricity generation. For these impact categories, the impact from exhaust emissions is minor, making MGO a better option. However, if metal emissions from the combustion are included, the toxicity impact may increase.^22^ For the BE concept (case 8), 35% of the human toxicity cancer effects, 30% of the human toxicity non-cancer effects, and 20% of the freshwater ecotoxicity are related to battery production. FC with cleaner electrochemical oxidation has a relatively low impact on all categories. For eMeOHICE w PostCC (case 2), the impact on all categories is higher compared to eMeOHICE (case 1), which is because more fuel is needed for the PostCC system. For impact categories mainly influenced by engine emissions (acidification, photochemical ozone formation, particulate matter, and eutrophication), the formation of NO*_x_* and NH_3_ slip has to be significantly reduced to reduce these impacts. A major uncertainty in this study is related to eNH3ICE, where full-scale tests of engines are needed to increase the knowledge of real NO*_x_*, N_2_O, and NH_3_ emission factors and to better understand the potential of using SCRs for exhaust abatement (where the NH_3_ slipped from the engine can act as a reducing agent for the NO*_x_* and N_2_O formed during combustion).

#### 3.2.3 Influence on GWP of Different Electricity Mixes

**Figure 6:**
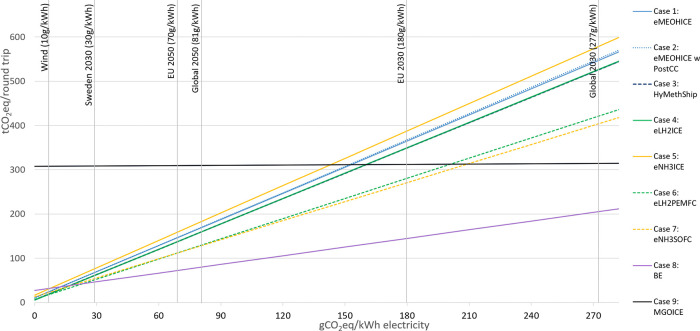
GWP100 based on LCA for different pathways considered in this study as a function of the carbon intensities of electricity used (0–300 kgCO_2_eq/kWh). The *x*-axis represents the carbon intensity of electricity.

The influence of the carbon intensity of the input electricity on the GWP of the assessed options is shown in Figure 6. There is a large influence on GWP for options with low energy conversion efficiency as more electricity is required. However, if the carbon intensity of the electricity mix is less than 150 gCO_2_eq/kWh, all non-fossil-fuel options will have a lower GWP than the reference case over the entire life cycle. In the scenario analysis for the BE option, the result shows that increasing the use of renewable electricity by storing it at the port using batteries can reduce the GWP100 by 20% for BE (SI S3.2), as shown in case 8c, whereas for case 8b (onboard batteries only for a single trip charged at the Swedish grid (port 1) and the German grid (port 2)), the GWP is 130% higher compared to the base case with battery for the round trip charged from the Swedish grid.

### 3.3 Economic Impact Assessment

**Figure 7:**
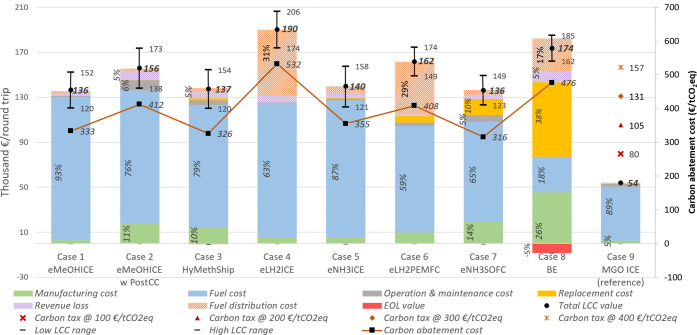
Economic assessment of different decarbonization options over the entire fuel life cycle in terms of eLCC also indicating the impact of uncertainty and scenario analysis and carbon abatement cost. The bars represent the mean value of costs associated with different phases, and the points represent the total eLCC with an uncertainty range from the Monte Carlo simulation, where the upper bound represents the 75 percentile and the lower bound represents the 25 percentile of 10,000 iterations. The carbon abatement cost is represented by black squares linked with a line and values in the secondary *y*-axis (right). The percentage contribution from different life cycle stages is also shown (less than 5% is not marked). The fuel distribution and revenue loss are parameters not included in LCA but added in the scenario analysis of eLCC. The effect on the MGO cost of different carbon taxes is also presented.

Figure 7 shows the eLCC results including the Monte Carlo simulation, the carbon abatement cost, and the estimated fuel distribution cost. None of the studied options are cost-competitive with the MGO options over the life cycle, and the carbon abatement cost (which also can reflect the carbon tax needed for the option to be cost-competitive compared to MGO) varies from about 300 to 550 €/tCO_2_eq (including the distribution cost and revenue loss). The effect on the MGO cost of different carbon taxes is also presented in Figure 7. With a carbon tax above 300 €/tCO_2_, the studied options start to become competitive. Except for eLH_2_ and BE, all options would be economically feasible with a carbon tax of 400 €/tCO_2_. eNH3SOFC has the lowest eLCC and carbon abatement cost. Excluding the fuel distribution cost, eLH2PEMFC has the lowest cost. For all concepts, except for BE, the major cost is associated with the fuel cost. Due to higher efficiencies of the fuel cell, the amount of fuel required for the round trip is lower for these cases, which makes the fuel cell options economically attractive. Fuel costs used in the study are calculated by including uncertainty parameters using Monte Carlo simulations, and the result shows similar uncertainty patterns for different fuels; see SI S4.1. For BE, the major cost is associated with investment and distribution costs, the former due to the short lifetime leading to multiple replacements. BE has the second-highest eLCC and abatement cost.

For methanol options, HyMethShip has a cost advantage over the other cases due to the higher CO_2_ recirculation, reducing the CO_2_ demand from DAC. PostCC is the most expensive option although the need for CO_2_ from DAC is reduced, and case 2 has the highest carbon abatement cost, which is due to increased fuel use, more consumables, and higher investment. In addition, the cost associated with the revenue loss from the extra space required for fuel storage is not a major cost for any of the cases. The cost of fuel cell options is lower than respective ICE options since the higher investment cost is offset by the reduced fuel demand. The Monte Carlo uncertainty assessment shows that the cost range varies between ±8% (for BE) and ±13% (for ICE options) for the changes made. The lower uncertainty for the BE option is because the cost of the energy carrier (here electricity) represents a lower share of the total cost (due to the high cost associated with the battery).

## 4 Discussion

In the case of electricity with relatively low carbon intensity, all of the assessed options can substantially reduce the GHG emissions of ships. However, the cost is 2.5–4 times higher than the MGO option. Also, the energy conversion losses for the fuel delivered are high and there would be an increased electricity demand. The GHG reduction for all of the options seems to come with some trade-offs in terms of other environmental impacts such as human toxicity (cancer and noncancer effects) and freshwater ecotoxicity, which are increased in this assessment in comparison to the reference case. These impacts are from the wind power infrastructure, which currently requires more building materials per energy output including minerals like copper, zinc, and rare earths in addition to steel compared to other electricity production infrastructures.^93^

The fuel distribution cost increases the cost for hydrogen options substantially, making eNH3SOFC the most cost-effective option for reducing GHG emissions. This is in line with Stolz et al.^38^ Horvath et al.^94^ found eLH2PEMFC to be a cost-effective choice (fuel distribution not included); however, Korberg et al.^14^ found electro-methanol to be a more cost-effective option. To the authors’ knowledge, there are no studies that have analyzed the eLCC and LCA for the post-carbon capture option. However, the higher cost of the battery-electric option is highlighted in Lloyd’s Register and UMAS,^11^ while Perčić et al.^18^ showed that battery-electric vessels can have a lower total cost than diesel-powered ships in the Croatian short-sea shipping sector (up to 30 nautical miles without considering the charging infrastructure). For shorter distances, the battery-electric option would be viable considering the lower battery capacity required onboard, which can be attained by increasing the battery charging frequency. However, it increases the number of charging and discharging cycles, which may result in added costs due to early battery replacements (more analyses are required). Lindstad et al.^10^ found the abatement costs for electro-hydrogen to be between 198 and 383 €/tCO_2_ (against this study 408 €/tCO_2_ for FC and 532 €/tCO_2_ for ICE), electro-ammonia between 152 and 344 €/tCO_2_ (against this study 316 €/tCO_2_ for FC and 355 €/tCO_2_ for ICE), and electro-methanol between 213 and 636 €/tCO_2_ (against this study 326–412 €/tCO_2_). Lindstad et al. did not consider revenue loss and fuel distribution cost. Korberg et al.^14^ showed the total cost of ownership compared with MGO between electro-hydrogen to be between 5 and 5.5 times for ICE (this study 3.5–4 times), between 4 and 6 times for FC (this study 3–3.5 times), electro-ammonia between 4 and 4.5 times for ICE (this study 2.5–3 times), between 4 and 6 times for FC (this study 2.5–3 times), and electro-methanol between 3.5 and 4.5 times for ICE (this study 2.5–3 times). Korberg et al. did not consider carbon capture options and also replacement cost, operation cost, and fuel distribution cost associated with the ship’s life cycle. Also, in this study, better performance data is used considering the likely development of these technologies. The introduction of market-based measures such as a carbon levy on fossil marine fuels has been discussed within IMO (levels of about 100 €/tCO_2_ have been proposed), but no agreement has been reached so far.^95,96^

From the cost, environmental, and energy utilization perspectives, FCs are shown to be more promising than ICEs for the same fuel despite the higher capital cost of FCs and their short lifetimes. The same observation was found in several studies;^38,94,97^ however, this is dependent on the hours per year the ship is in operation, where according to^14^ less operating hours per year lead to FCs being less competitive than ICEs. The electrical propeller used in battery-electric and FCs can offer better hydrodynamic efficiency,^98^ which can further increase the operational efficiency. However, the fuel cells and batteries may degrade over time, which can affect the efficiency of the system.

For ICE options, methanol and ammonia combustion have a significant impact on impact categories like acidification, marine eutrophication, photochemical ozone formation, and particular matters due to the tailpipe emissions of nitrous oxide, nitrogen oxides, and ammonia, as well as emissions of carbon compounds (for eMeOHICE). Hydrogen in ICE is expected to have cleaner combustion; hence, eLH2ICE and HyMethShip options have a lower impact on these environmental impact categories. This reduction potential of HyMethShip has been highlighted by Malmgren et al.^22^ eNH3ICE, eLH2ICEs, and eNH3SOFCs are in a very early stage of development, which means that the input data is more uncertain, and more updated emission and performance data (as the technologies develop) would increase the robustness of the results. The uncertainty in climate change impact is particularly high for eNH3ICE due to uncertainty in likely nitrous oxide emissions.

This study includes heat pumps for low-quality heat. Only a few studies have looked into the feasibility of heat pumps onboard ships,^99^ and a detailed study of heat integration may help optimize energy use further. Also, high deployment of assessed technologies is set to drive the increased requirement for minerals and renewable electricity, and future studies may be conducted to analyze the role of resource criticality and recycling utilization from a life cycle perspective at the fleet level. Future studies may include other electro-fuels, particularly in fuel cells in addition to port infrastructure and additional emissions to water and soil as well as improved knowledge on emissions from ammonia fuel cells and engines. Detailed ship designs are also needed to better understand the impact on the payload of the increased weight and volume of the propulsion systems. In addition, improved cost estimates should be considered, along with more detailed assessments of the need and costs for the distribution and storage of energy carriers. These are not included in the result as the fuel cell technologies are still in the development phase and inventory data are not available. Similarly, there is not much data on the port infrastructure and emissions to water and soil during ship operation for the alternate fuels. Data is key for such analyses. The development of the shipping sector depends on more aspects than the economic and environmental performance of the systems, and the future choices made by stakeholders are complex. This study has, for example, not taken into account how parameters like acceptance, handling safety, energy security, and employment creation will influence the choice of decarbonization pathways, but social assessments of future fuels are also important.

To conclude, the study gives a detailed assessment of different decarbonization pathways from a life cycle perspective in terms of energy requirement, environmental impact, and cost. The options could be used to meet IMO GHG reduction targets; however, the cost over the life cycle increases by a factor of 2.5–4, and the largest part of the cost is associated with the fuel except for the battery-electric case. From a policy perspective, the carbon abatement cost is a relevant measure indicating the cost associated with GHG removal. The carbon abatement cost for the studied options ranges from 300 €/tCO_2_eq to 550 €/tCO_2_eq, with the lowest being ammonia in fuel cells, closely followed by the HyMethShip concept and methanol in ICE. These concepts are based on the anticipated likely development, and the results show only the potential implication of technology. As the EU carbon price has not yet passed 100 €/tCO_2_ and is not expected to do so by 2030,^100^ this implies that major incentives and policy measures are required to promote GHG reductions for shipping linked to fuel shift.

## Supporting Information Available

Additional data and results of the article; details of the methodology, background, and case studies (Section S1); details of the inventory data used for the analysis (Section S2); life cycle assessment results including sensitivity analysis and uncertainty analysis (Section S3); and fuel cost analysis and life cycle cost results (Section S4) (PDF)
