# Supplementary material for: Life-Cycle Assessment and Costing of Fuels and Propulsion Systems in Future Fossil-Free Shipping
Source: Environ Sci Technol. 2022 Aug 23;56(17):12517–31. doi: 10.1021/acs.est.2c03016 (PMC9454245; doi:10.1021/acs.est.2c03016)
Supplement: Supplementary file 1 — es2c03016_si_001.pdf [file es2c03016_si_001.pdf]

Supplementary information

Life Cycle Assessment and Costing of Fuels and Propulsion Systems in Future Fossil-Free Shipping

Fayas Malik Kanchiralla[1*], Selma Brynolf[1], Elin Malmgren[1], Julia Hansson[1 2], Maria Grahn[1]

[1]Chalmers University of Technology, Department of Mechanics and Maritime Sciences, Chalmersplatsen 4, SE-412 96, Gothenburg, Sweden

[2]IVL Swedish Environmental Research Institute, Aschebergsgatan 44, SE-411 33, Göteborg, Sweden

*corresponding author: fayas.kanchiralla@chalmers.se ; *+46 31 7721439*

This supplementary information provides all background information to the publication and covers details on the methods, case study, technical description, background materials, motivation on the assumption, inventory data, and detailed supplementary results used in this study. The SI also includes detailed results like results before normalization, uncertainty analysis, and scenario analysis used in this study. The document contains 34 pages, 28 Figures, and 14 Tables.

Table of Contents

S1: Applied methodology and detailed description 1
S1.1 Functional unit and time horizon 2
S1.2 Case study vessel details 2
S1.3 Technological options 3
S1.4 LCC methodology 6
S1.5 Limitations 7
S2: Inventory details 8
S2.1 Fuel properties 8
S2.2 Fuel production phase 8
S2.3 Component manufacturing and end of life 10
S2.4 Operation phase 13
S2.5 Cost details 15
S2.6 Datasets 17
S3: Life cycle assessment result 18
S3.1 Environmental impact assessment 18
S3.2 Scenario analysis 21
S3.3 Uncertainty analysis 21
S4: Life cycle cost results 26
S4.1 Fuel cost uncertainty 26
S4.2 Uncertainty analysis with port infrastructure 26
References 27

List of Figures

Figure S1:Methodological steps used in the study in line with ISO 14044:2006 for both pLCA and eLCC....4
Figure S3:Model showing the propulsion system for cases 4 to 6. 7
Figure S4:Model showing the propulsion system for cases 7 to 9. 7
Figure S5:System boundaries are defined in the study including background, and foreground systems for eLCC. The inventory cost flow is different from the material and energy flows in the pLCA. 8
Figure S6:pLCA results of different propulsion options for impact category acidification for the round trip. 20
Figure S7:pLCA results of different propulsion options for impact category freshwater ecotoxicity for the round trip. 20
Figure S8:pLCA results of different propulsion options for impact category marine eutrophication for the round trip. 21
Figure S9:pLCA results of different propulsion options for impact category terrestrial eutrophication for the round trip. 21
Figure S10:pLCA results of different propulsion options for impact category human toxicity, cancer effects for the round trip. 21
Figure S11: pLCA results of different propulsion options for impact category human toxicity, non-cancer effects for the round trip. 22

Figure S12: pLCA results of different propulsion options for impact category ozone depletion for the round trip. 22
Figure S13: pLCA results of different propulsion options for impact category particulate matter for the round trip. 22
Figure S14: pLCA results of different propulsion options for impact category photochemical ozone formation for the round trip. 23
Figure S15: Climate change impact for different scenarios for BE option. 23
Figure S16:Monte-Carlo simulation results of different propulsion options for impact category climate change GWP100. 24
Figure S17:Monte-Carlo simulation results of different propulsion options for impact category climate change GWP20. 24
Figure S18:Monte-Carlo simulation results of different propulsion options for impact category acidification for the round trip. 25
Figure S19:Monte-Carlo simulation results of different propulsion options for impact category freshwater ecotoxicity. 25
Figure S20: Monte-carlo simulation results of different propulsion options for impact category marine eutrophication. 25
Figure S21:Monte-Carlo simulation results of different propulsion options for impact category eutrophication terrestrial. 26
Figure S22:Monte-Carlo simulation results of different propulsion options for impact category human toxicity, cancer effect. 26
Figure S23:Monte-Carlo simulation results of different propulsion options for impact category human toxicity, non-cancer effect. 26
Figure S24: Monte-Carlo simulation results of different propulsion options for impact category ozone depletion. 27
Figure S25:Monte-Carlo simulation results of different propulsion options for impact category particulate matter. 27
Figure S26:Monte-Carlo simulation results of different propulsion options for impact category photochemical ozone formation. 28
Figure S27:Cost of the fuel calculated and used for the calculation of LCC. The uncertainty range is based on the Monte-Carlo analysis. 28
Figure S28:eLCC results including the cost associated without the fuel distribution. 29

Table of Figures

Table S1:Main particulars of the case study vessel including the energy load during the entire round-trip operation. 5
Table S2:Properties of the fuels considered in this study. 10
Table S3: Electricity production scenario for the year 2030 and the LCI data used for the different energy scenarios. 10
Table S4:LCI background data used for the fuel production infrastructure. 12
Table S5: The material and energy details of the marine engine considered in the study. 12
Table S6:The material and energy details of the PEMFC considered in the study. 13
Table S7:The material and energy details of the SOFC considered in the study. 13
Table S8:The material and energy details of the reformers considered in the study. 15
Table S9: Materials required for the heat pump considered in the study. 15

Table S10: Consumables required for PostCC operation and emissions during the operation.....17
Table S11: The scrap cost of the material during the end of life. 17
Table S12: The volume and weight of the propulsion system for the cases considered in the study. 18
Table S13: Fuel distribution cost used for the study...18
Table S14:Materials considered in the manufacturing of different components and relation to the database. 19

S1: Applied methodology and detailed description

In line with the objective of the energy performance, environmental performance, and economic performance are investigated from the life cycle perspective including all life cycles from cradle to grave of a product or service. The prospective life cycle assessment (pLCA) and environmental life cycle costing (eLCC) are methodologies used in this study for environmental and economic assessment taking care of their entire life cycle from cradle to grave. The standardized guidelines of ISO 14044:2006 standard are used in four phases: 'goal and scope definition', 'inventory analysis', 'impact assessment', and 'interpretation' in an iterative process [1]. The technologies assessed in the option are in the early stages of development and need to be modeled at a future point in time, and should include the likely development in the goal, scope, and inventory modeling. Future-oriented LCA studies also called ex-ante LCA is used for the assessment of emerging technologies or consequences of the introduction of new technologies. The pLCA comes under the scope of ex-ante LCA is used [2] (terminology used in different literature is different). emerging technology is scaled-up to include technology development and using likely performance at full operational scale [2, 3]. The midpoint level is used in this study as a higher number of impact categories and the results are more accurate and precise compared to the three areas of protection commonly used at the endpoint level [4].

Life cycle costing (LCC) is a tool for assessing the economic dimension of sustainability and is capable to support decision-making at different stages of the life cycle [5]. Rödger et al. [5] distinguish LCC into conventional LCC, environmental LCC, and societal LCC. The eLCC method is aligned with the LCA study in terms of system boundaries, functional units, and methodological steps [5]. In LCC, analysis is done based on cost flows in terms of expenses (outflow) and revenue (inflow) over different life cycle phases. In eLCC, environmental emissions or wastes from the system would also be internalized in terms of monetary value. In this study, sensitivity analysis is done assuming an anticipated carbon tax on fossil-based CO_2 emissions. Another aspect of LCC is that there are several stakeholders involved in different life cycle phases and each stakeholder has a different type of impact. For aggregating the cost simply adding the costs of all actors in the life cycle will not work as the expense of one actor is the revenue of another [5]. The LCC can be performed for multiple actors, however, this study focuses on ship owners and operators. For example, the costs flow associated with manufacturing components (energy costs, material costs, transportation, etc.) are expenses to the manufacturer whereas the acquisition cost is revenue. Figure 1 shows the main steps of the pLCA and eLCC methodology.

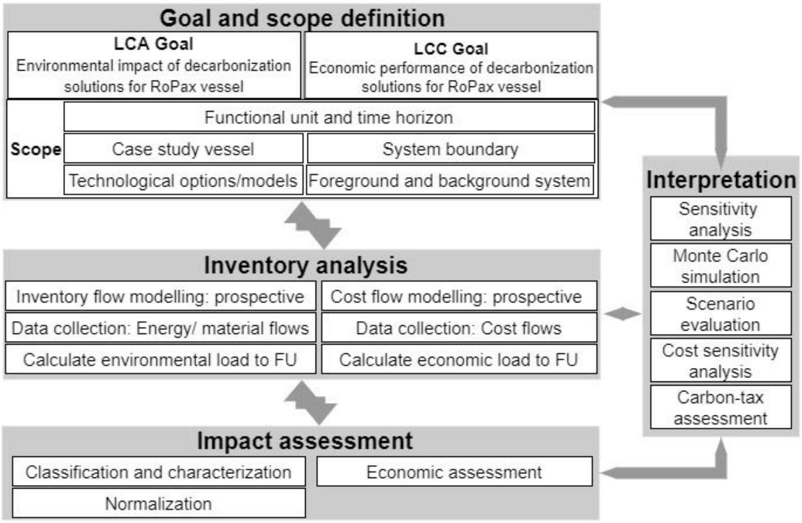

Figure S1:Methodological steps used in the study in line with ISO 14044:2006 for both pLCA and eLCC

S1.1 Functional unit and time horizon

In eLCC and pLCA, the functional unit is key to comparing the cases and should be a quantifiable reference flow representing the product system's function [1]. The functional unit used in the study is *one round trip of a RoPax vessel from Gothenburg to Kiel and back to Gothenburg*. The pLCA studies emerging technologies when the knowledge is sparse [3] and considers the environmental performance at a time in the future when the technology is likely to get developed. It is assumed that the technologies are developing at a faster pace in recent years and would be matured by end of this decade, the time horizon considered for this study is 2030, these solutions will be get developed within 10 years and would be matured by end of this decade. Further, it is assumed that the ship operates for 25 years before the ship is disposed of. The study is made in a European context, and targeted audience includes ship owners, operators, academia, policymakers, and LCA practitioners**.**

S1.2 Case study vessel details

The vessel considered in this study is traveling on a fixed route of roughly 230 nautical miles between Gothenburg (port 1) and Kiel (port 2). Round trip energy data is calculated using the data measured from the cruising phase, maneuvering phase, and Mooring phase of a single trip, and hotel load is assumed as 75% of maximum load. The main particulars of the case study ship are shown in Table S1. The propulsion system for each case study is designed based on the reference ship's voyage route and operation profile considering the maximum propeller load of 20MW, maximum hotel load of 2 MW, the maximum auxiliary load of 2 MW, and the additional energy loads required for the operation of components like reformer, onboard carbon capture, etc., and to compensate for the energy losses during the operation of components.

Table S1:Main particulars of the case study vessel including the energy load during the entire round-trip operation.

Vessel type	RoPax Ferry		Energy load of the ship during the round trip		
Route	Gothenburg - Kiel		Cruising	Propeller mechanical load (kWh)	382 500
Length	240 m			Auxiliary electrical load (kWh)	31 600
Deadweight	10130 MT			Thermal heat load (kWh)	31 200
Design speed	19 knots		Maneuvering	Propeller mechanical load (kWh)	22 400
Passenger capacity	1300			Auxiliary electrical load (kWh)	5 800

Vehicle capacity	300			Thermal heat load (kWh)	5 000
Annual round trips	182		Mooring	Port 1 -electrical (kWh)	12 000
Service Life	25 years			Port 2 -electrical (kWh)	9 800

S1.3 Technological options

In this study, the following nine propulsion options that also need to meet Tier III requirements are analyzed from a life cycle perspective. These propulsion scenarios are modeled by selecting major components and their likely development by 2030, In addition to the load profile of the ship, the component size and capacity also depend on the additional loads, the efficiencies of the other associated components, and the availability of excess heat from the engine. During the mooring phase, the only electrical load is considered and is assumed from the respective port uniformly for all the cases. Reserve capacity for the storage tank is assumed as 30% of the total fuel required for the round trip.

Case 1 MeOHICE is a propulsion option fueled by eMeOH in a dual-fuel engine with SCR and the pilot fuel is MGO (Figure S2). The total engine power required for the operation is 21 MW. The shaft generator is required only to meet the auxiliary electrical load. The excess heat from the engine is used with help of waste heat recovery (WHR) to meet the heat requirement during cruising and maneuvering. eMeOH and MGO are stored in normal storage tanks.
Case 2 MeOHICE w PostCC is a dual fuel engine fueled by eMeOH with post-combustion OCCS and the pilot fuel is MGO (Figure S2). The total engine power required is 25 MW due to the higher energy required for the additional component. The shaft generator is required for meeting the auxiliary load and additional energy for the heat pump, OCCS, and CO_2 chiller. eMeOH and MGO are stored in service tanks and liquid CO_2 is stored in insulated cryogenic tanks. Excess heat from the engine is used in OCCS operation. The CO_2 captured onboard is circulated back for eMeOH production onshore.
Case 3 HyMethShip which is detailed in the study by Malmgren et al. [6]. In this case, eMeOH is used as an energy carrier and stored onboard (Figure S2). The eMeOH is cracked in a reformer into H_2 and CO_2. CO_2 is separated with the help of membranes in the reformer. The H2 is burned in the 25 MW spark-ignition engine coupled with a generator. Electric propulsion is considered to meet the torque requirement. The engine is optimized for producing high-quality heat to use in the reformer and CO_2 adsorption chiller. For startup and power ramping a 5 MWh battery storage system is considered. The NOx emissions from engines are below the Tier III requirement [6]. The eMeOH is stored in service tanks and liquid CO_2 is stored in insulated cryogenic tanks. The CO_2 captured onboard is circulated back for eMeOH production.

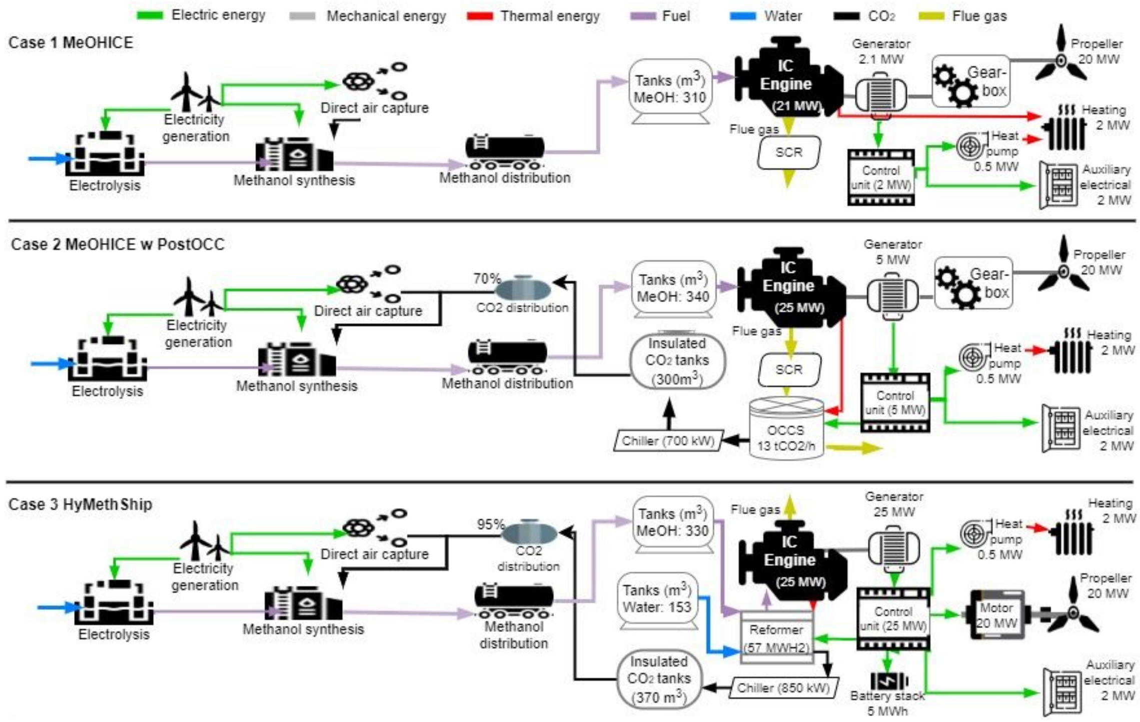

Figure S2: Model showing the propulsion system and fuel production for cases 1 to 3.

Case 4 eLH2ICE is H_2-fueled in a spark-ignition engine, where eH2 is stored as liquid (eLH2) (Figure S3). The engines are coupled with the generators with a total power output of 24 MW and SCR is not considered. eLH2 must be stored below -253°C and require special vacuum insulation tanks. The excess heat from the engine is used with help of WHR to meet the heat requirement.

Case 5 eNH3ICE is a propulsion option fueled by eNH_3 in spark-ignition engines which are coupled with the generators with a total power output of 24 MW (Figure S3). The electricity for the heat pump and auxiliary load also comes from these generators. The system uses H_2 as pilot fuel which is generated using an NH_3 reformer operated from excess heat from the engine. The system uses a 5 MWh battery storage system for startup and power ramping. SCR system is required for reducing NOx emissions, SCR in this system can utilize NH_3 from the engine slip as a reducing agent. NH_3 storage can be stored as a liquid when pressurized above 18 bar [7] and is stored in pressurized storage tanks.

Case 6 eLH2PEMFC is powered using PEMFC fueled by H_2, where eH_2 is stored as eLH2 (Figure S3). The total power output of 24 MW. Since the energy output is electrical, it can be directly used for the electrical propulsion, heat pump, and auxiliary load. eLH2 is stored in special vacuum insulated tanks below -253 ^{0}C. PEMFC is the most commercialized fuel-cell type and operates with high purity H_2 to avoid electrolyte/electrode poisoning [8].

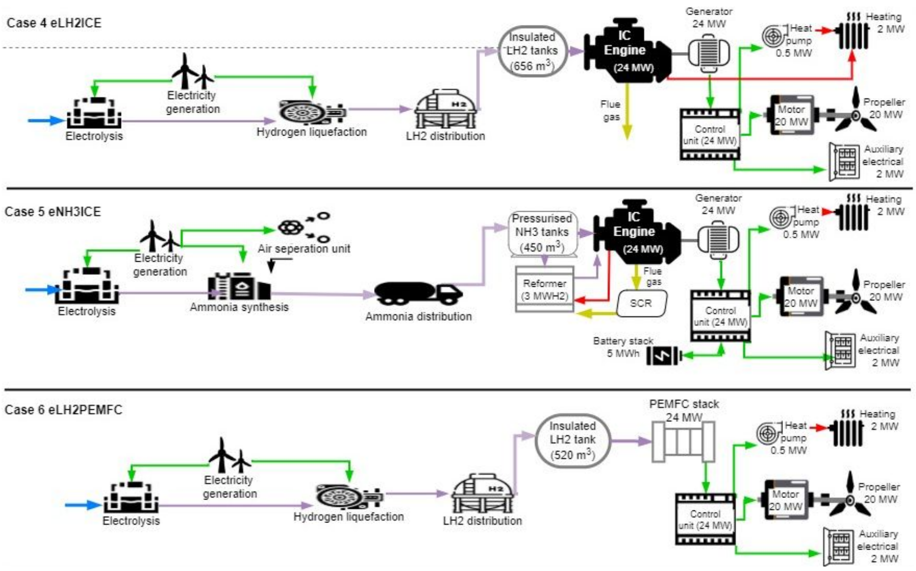

Figure S2:Model showing the propulsion system for cases 4 to 6.

Case 7 eNH3SOFC is powered using SOFC fueled by eNH_3 with a total power output of 23 MW (Figure S4). The electrical output is directly used for electrical propulsion, heat pump, and auxiliary load. eNH_3 is stored as a liquid in tanks pressurized above 18 bar. For startup and power ramping the system uses a 5MWh battery storage system. SOFCs are selected for NH3 as i) Zirconia-based solid electrolyte is stable in NH3, ii) High temperature allows internally reforming of NH3 into H2 and nitrogen with help of a catalyst, and iii) The excess heat generated from the chemical reaction can be utilized for NH3 reforming [9].
Case 8 BE, where the electricity is stored in batteries and used for the ship operation and is charged using electricity from the port (Figure S4). The battery is sized for a round trip with a capacity of around 700 MWh including a 30% reserve capacity. Power is managed using the control unit and is directly used for electrical propulsion, heat pump, and auxiliary loads.
Case 9 MGOICE is the reference case where fossil MGO is fueled in a conventional medium-speed diesel engine is the reference case (Figure S4). The total engine power required for the operation is 21 MW with SCR. The shaft generator is required for meeting the auxiliary electrical load. The excess heat from the engine is used with help of WHR to meet the heat requirement.

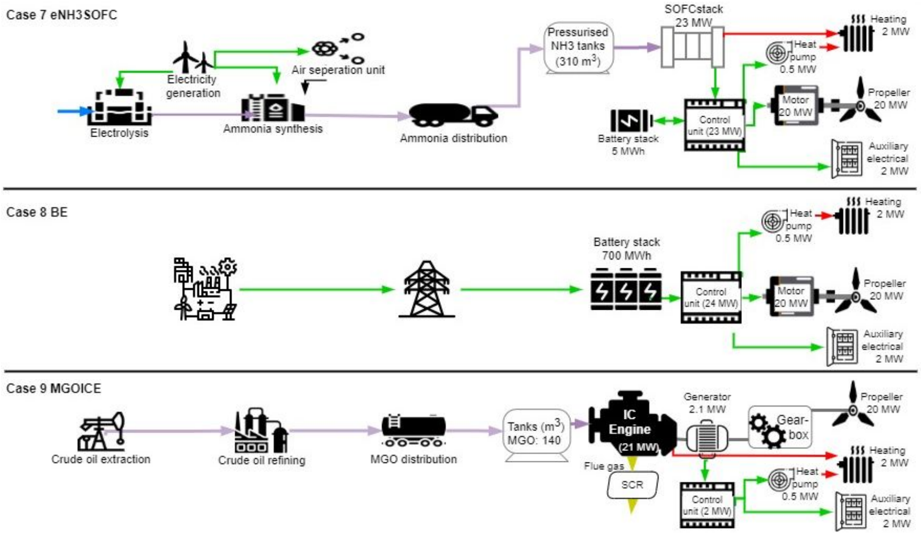

Figure S3:Model showing the propulsion system for cases 7 to 9.

S1.4 LCC methodology

The cost flows around the processes are different from pLCA and so is the system boundary for foreground system and background system as shown in Figure S5, the round-trip cost constitutes cost from different life cycle phases and the cost is incurred in different time frames and period. The cost flows are considered in Euros (€) with the base year 2021 and are estimated for the year 2030. The number of annual trips (t_a) is 182 and the service life of ship (n) and infrastructure is considered for 25 years. Capital recovery factor (crf) is considered for fuel infrastructure and capital equipment, with a discounting rate of 3%.

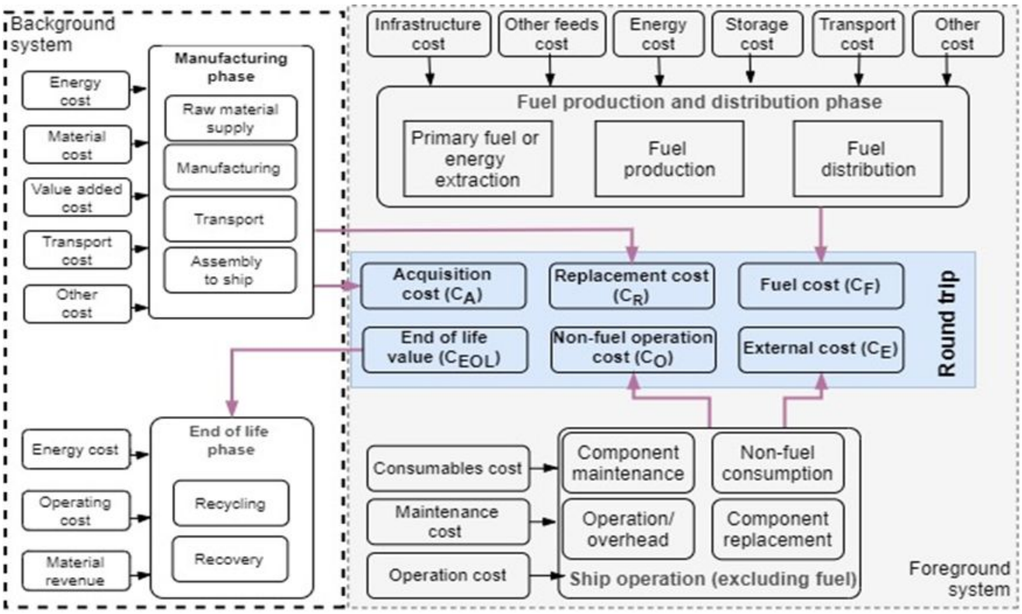

Figure S4:System boundaries are defined in the study including background, and foreground systems for eLCC. The inventory cost flow is different from the material and energy flows in the pLCA.

Manufacturing phase: The cost of each component is decided based on the sizing and rating, which are detailed in Table 2 and Table 3 for each propulsion system option. The total equipment cost (TEC) of a given propulsion system is calculated by adding the cost of all the components required for the given system. Equation 2 is used to convert the purchase cost towards the round-trip termed as acquisition cost (C_A).

$$C_A(€/round\ trip) = \frac{TEC \times crf}{t_a} \quad (2)$$

Fuel production phase: The energy carriers selected for this study are fossil-based MGO, fossil-based LNG, eMeOH, eLH_2, eNH_3, and electricity mix costs are calculated as part of the study considering all the input prices. MGO and LNG are traded presently in the market and major changes are not expected by 2030 in terms of the production processes except due to supply and demand. For these fuels, the cutoff price is taken from the 2021 yearly average. For the other alternative fuels, the fuel cost (F_C) (in €/MWh_{fuel}) is calculated by considering all the upstream costs (details in supporting information section S2.2). The fuel cost for the round trip (C_F) is dependent on the amount of fuel consumed in the round trip (F_R) as in equation 3.

$$C_F(€/round\ trip) = F_C\ (€/kg) \times F_R(kg) \quad (3)$$

The replacement cost: Some capital equipment needs to be replaced due to the lesser operational life of the component compared to the service life of the ship (e.g. batteries). Replacement cost is

different for each component and is based on the number of replacements (Nr); all replacements required for propulsion to be summed to get the total cost. The replacement cost for the round trip (C_R) is calculated using equation 4, where Nc is the number of components that require replacement, and Rc is the cost for each replacement.

$$C_R = \sum_1^{Nc} \frac{Nr \times Rc \times crf}{t_a} \quad (4)$$

Operation phase: During the operation life of the ship there are different costs associated with the consumables (C_C) (e.g.: urea used in SCR, MEA used in OCC, etc.), operation/overhead cost (C_O), and service/ maintenance cost (C_M). The prices for the consumables are determined by market prices and are directly taken into the calculation. The operation cost excluding the replacement cost and fuel cost for the round trip is termed C_{OMC} in the study and calculated using equation 5.

$$C_{OMC} = \frac{C_O}{t_a} + \frac{C_M}{t_a} + C_C \quad (5)$$

EOL phase: The EOL phase is considered in the background system, only the scrap values for the components are analyzed. This depends on the material composition of the components. In this analysis the scrap value for the metals (the reference year 2021) for calculating the total value that can be recovered. The material will get recovered only at the end of the life of the component, considering that there would be inflation in the material price. The discount rate is not used in this case. End-of-life cost for the round trip (C_{EOL}) is treated as revenue in this study and calculated using equation 6.

$$C_{EOL} = \frac{Amount\ of\ metal\ in\ component\ (kg) \times scrap\ value\ of\ metal\ (€/kg)}{t_a \times n} \quad (6)$$

External cost: The CO_2 emitted during fuel consumption is expected to be internalized by 2030. In this study, only the CO_2 emission is used to calculate the environmental costs. The internalized environmental cost (C_E) for operation phase emissions is calculated in this phase in addition to the cost for CO_2 transportation for permanent storage (equation 7). This study uses 150 €/tonne of CO_2 as the anticipated carbon tax during 2030 [10].

$$C_E(€/round\ trip) = Tax\ rate\ (€/kg) \times emissions\ during\ round\ trip\ (kg) \quad (7)$$

Revenue loss: Due to differences in the volumetric efficiency of the fuel, power densities of different energy conversion technologies, and additional components like CC, reformer, etc. additional volume is required on-board of ships. For ferries, volume loss is considered more important than tonnage loss and space loss cost (C_{SL}) is assumed at 8 €/m^3 as in the study by Korberg et al.[11]. The revenue loss (C_{RL}) is calculated using equation 8, where V_A is the additional volume required.

$$C_{RL}(€/round\ trip) = C_{SL}\ (€/m^3) \times V_A\ (m^3) \quad (8)$$

Finally, the total life cycle cost for the round trip (functional unit) including all the life cycle phase costs for the round trip of case RoPax vessel is shown in equation 9:

$$Total\ life\ cycle\ cost\ (€/round\ trip) = C_A + C_F + C_{OMC} + C_R + C_E + C_{EOL+}\ C_{RL} \quad (9)$$

S1.5 Limitations

Due to the different volumetric and gravimetric energy densities of the fuel/battery, the volume and weight of the fuel tanks onboard would be different. Also, the power density of ICEs and FCs varies with different options. The potential impact on the ship operation due to the difference in

weight/volume of these systems is not addressed in this study. Another limitation of the study is regarding the system boundary, that the scope does not include the port infrastructure required for each fuel type nor the transportation of fuels from the production facility to the port. Considering that the ship structure and components other than the propulsion/fuel system would be the same for all the cases, it is not considered in the study. Although a higher amount of charging and discharging cycle has a negative effect on the battery life, charging cycles for the batteries are not considered in the study.

S2: Inventory details

S2.1 Fuel properties

The properties of the fuels studied are given in Table S2.

Table S2:Properties of the fuels considered in this study.

	Liquid Ammonia [12]	*Liquid Hydrogen [12]*	*Methanol[13]*	*MGO*
Boiling point [7]	-33.4	-253	65	200-385
Lower heat value [MJ/kg]	18.8	120	19.9	42.7
Auto ignition temperature [^oC]	651	571	439	250
Flammability Limits in air [vol.%]	15-28	4.7-75	7.3-36	0.5 -5
Density (kg/m^3)	682.6	70.8	791.4	855

S2.2 Fuel production phase

MGO is the only fossil-based fuel considered in the study which is considered a background process and inventory details are taken from the Ecoinvent 3.7.1 (diesel production, low-sulfur, petroleum refinery operation | diesel, low-sulfur | Cutoff).

Electricity is used for four applications, i) fuel production/liquefaction, ii) component production, iii) mooring at Gothenburg port (both for energy use/charging batteries), and iv) mooring at Kiel port (both for energy use/charging batteries). For each application, the source of electricity varies and has different associated costs and emission factors. For fuel production of eLH_2, eNH_3, and eMeOH, the renewable electricity considered in the study is from onshore wind power with LCI data from Ecoinvent 3.7.1. For the component production application, electricity is assumed from the European electricity mix as the component is assumed to be produced in different locations in Europe. During mooring at Gothenburg port, the Swedish electricity mix is considered. At Kiel port, during mooring German electricity mix is considered. Electricity mixes are based on projected scenarios for the time horizon 2030 by the EU Commission based on the reference year 2020 and present member policy [14] as shown in Table S3.

Table S3: Electricity production scenario for the year 2030 and the LCI data used for the different energy scenarios.

	EU mix [14]	*Swedish mix [14]*	*German mix [14]*	*Description of the LCI database*	*Reference*
Nuclear energy	17.3%	28.3%	0.0%	electricity production, nuclear, pressure water reactor | electricity, high voltage | Cutoff	Ecoinvent 3.7.1
Solids	9.0%	0.0%	20.4%	electricity production, hard coal | electricity, high voltage | Cutoff	Ecoinvent 3.7.1
Oil	0.2%	0.0%	0.2%	electricity production, oil | electricity, high voltage | Cutoff	Ecoinvent 3.7.1
Gas	14.5%	0.8%	15.7%	electricity production, nuclear, pressure water reactor | electricity, high voltage | Cutoff	Ecoinvent 3.7.1

Biomass waste	5.7%	9.8%	7.9%	heat and power co-generation, biogas, gas engine | electricity, high voltage | Cutoff	Ecoinvent 3.7.1
Hydro-lakes	6.4%	14.9%	0.4%	electricity production, hydro, reservoir, non-alpine region | electricity, high voltage | Cutoff	Ecoinvent 3.7.1
Hydro-run of water	5.7%	25.7%	3.6%	electricity production, hydro, run-of-river |electricity, high voltage | Cutoff	Ecoinvent 3.7.1
Wind-onshore	22.5%	19.0%	23.8%	electricity production, wind, >3MW turbine, onshore | electricity, high voltage | Cutoff	Ecoinvent 3.7.1
wind offshore	6.8%	0.4%	13.4%	electricity production, wind, 1-3MW turbine, offshore | electricity, high voltage | Cutoff	Ecoinvent 3.7.1
Solar	11.7%	1.1%	14.7%	electricity production, photovoltaic, 570kWp open ground installation, multi-Si | electricity, low voltage | Cutoff	Ecoinvent 3.7.1
Geothermal and other renewables	0.2%	0.0%	0.0%	electricity production, deep geothermal | electricity, high voltage | Cutoff	Ecoinvent 3.7.1

Hydrogen can be produced from different feedstock and presently over 95% of the current hydrogen is produced from fossil fuel-based energy sources [15]. The study focuses on **eH_2** which is produced by electrolysis of water using renewable energy. The study from Delpierre et al. [16] suggests that there is no significant variation in environmental impact between the PEM electrolyzer and the alkaline electrolyzer. As the alkaline electrolyzer is more mature and less expensive, this study only considers alkaline electrolyzer. This study uses inventory data for an alkaline electrolyzer from the LCA study by Delpierre et al. [16]. The production of 1 kg of hydrogen requires 50kWh of electricity, 10 kg of water, and 1-2 g of potassium hydroxide [16]. Another possibility is to use a solid oxide electrolyzer, however, the cost of solid oxide electrolyzers is high that it is not cost-competitive with other electrolyzers [17] and hence not considered in the study. The LCI data for the electrolyzer is assumed from the study by Delpierre et al. [16].

Liquid Hydrogen is produced by liquefying the hydrogen whose boiling point is -253°C, theoretically, a minimum of 3.3 kWh of energy is required for liquifying 1 kg of hydrogen [15]. The results from the IDEALHY project show that the liquefication of H_2 can be achieved with 6.4 kWh/kg using a reverse Brayton cycle [18] which is assumed for this study considering that it would achieve commercial operation by 2030. For sensitivity analysis, energy for liquefaction is considered from 6 kWh/kg to 7 kWh/kg [15] (main article Table 5). The LCI and cost for the hydrogen liquefaction plant infrastructure are assumed from the IDEALHY project [18].

Methanol is also presently produced mainly from fossil fuels and can be produced from different feedstock including biomass and renewable energy sources [6]. **eMeOH** is produced using renewable energy, eH_2, and captured CO_2. For cases 2 and 3 the major share of CO_2 comes from capturing CO_2 from the onboard ship depending on the capture rate. The remaining CO_2 is considered from DAC based on the solid sorbent process. Leakage during the storage and transportation of CO_2 is assumed to be 1%. For case 5, it is considered that the entire CO_2 required comes from DAC. In this study, it is considered that producing 1 kg of eMeOH requires 1.375 kg of carbon dioxide, 0.189 kg H_2, and 0.858 kWh of electricity and heat [19]. During MeOH synthesis some byproducts get emitted to the air: CO_2 (7.65E-6 kg/kg MeOH), CO (6.9E-7 kg/kg MeOH), and MeOH (0.00172 kg/kg MeOH) [19]. The DAC technology used in commercial-scale DAC plants operated by Climeworks in Hinwil and Hellisheiöi is by cyclic temperature-vacuum swing adsorption and LCA has been conducted by Deutz and Bardow [20] for this plant. The same is considered in this study. The DAC plant encompasses the foundation, CO_2 collectors, process unit, auxiliaries, and the adsorbent [20]. Considering the time horizon of this study, the operation of the plant requires 3g adsorbent per kg CO_2 captured, 0.5 $kWh_{el,}$ and 1.5 kWh_{th} [20]. The thermal energy required for the process is assumed to be produced using electricity, hence the electricity

input is taken instead of the heat input. The LCI and cost for the eMeOH synthesis infrastructure are taken from Ecoinvent 3.7.1 and DAC infrastructure is considered from the study by Deutz and Bardow [20].

Presently **ammonia** is produced using Haber Bosch plants using fossil feedstocks – natural gas (50%), oil (31%), and coal (19%) [21]. This study focuses on **eNH_3** and there are multiple pathways for producing it such as the Haber Bosch process (TRL 9), electrochemical process (TRL 1-3), photocatalytic (TRL 1-3), biological (TRL 1-3), and non-thermal plasma (TRL 1-3) [21]. In this study, it is considered that eNH_3 is produced using the Haber Bosch process using eH_2 and nitrogen. The production of 1 kg eNH_3 requires 0.177 kg eH_2, 0.823 kg nitrogen, and 0.472 kWh of electricity [21]. From this process, the output is liquid NH_3. Nitrogen can be obtained with an air separation unit, cryogenic air distillation is a low-cost technology that can deliver high purity nitrogen in high volumes [22]. For separating 1 kg of nitrogen through cryogenic air distillation, 0.314 kWh of electricity is required [22]. NH_3 can be stored as a liquid above 18 bar pressure [7]. The LCI and cost for the eNH_3 synthesis and ASU infrastructure are taken from Ecoinvent 3.7.1.

The study includes the fuel production infrastructure required to produce the above fuels. These fuel production facilities are considered to be located near the Gothenburg port, this reduces transportation and additional storage requirements, and the fuel distribution and bunkering infrastructure are not considered in the study. The LCI detail and cost of the infrastructures considered in this study are given in Table S4.

Table S4:LCI background data used for the fuel production infrastructure.

Infrastructure	Description	Ref	Service life	CAPEX	O&M cost	Ref
Hydrogen liquefaction plant	Hydrogen liquefaction plant based on reverse Brayton cycle	[23]	25	2100 €/kgLH2/day	4%	[23]
Direct air capture plant	Direct air capture plant based on temp-vacuum swing adsorption	[20]	30	271 €/kgCO2/day	5%	[24]
Electrolysis plant	Alkaline electrolysis plant	[16]	30	570 €/kW	5%	[17]
Electro-methanol plant	Methanol factory construction | methanol factory | Cutoff, S	Ecoinvent 3.7.1	30	69 k€/tMeOH/day	5%	[17]
Air separation unit	air separation facility construction | air separation facility | Cutoff	Ecoinvent 3.7.1	30	376 €/kgN2/day	5%	[22]
Ammonia plant	chemical factory construction, organics | chemical factory, organics | Cutoff	Ecoinvent 3.7.1	30	174 k€/tNH_3/day	5 %	[17]

S2.3 Component manufacturing and end of life

Engine and SCR: During the engine manufacturing phase, the major emission is associated with the raw material and energy. The typical raw material composition used in the study is used from the study by Joeng et al [25] is shown in Table S5. The construction and weight depend on the type of engine which varies with the fuel used, the weight details for the MGO engine and MeOH DF engine are adopted from the manufacturers' catalog based on the power rating [26]. Selective catalytic reduction (SCR) is used for engines operated with MGO, eMeOH, and eNH_3. However, SCR requires high exhaust gas temperature and the addition of Urea or NH_3 for efficient reduction. If SCR is available, the engine can operate on a high fuel-efficient setting often associated with higher NOx from the engine (before SCR) [27]. This ensures that there is no major loss of efficiency. The activating element on the catalyst is assumed as TiO2 and is around 0.25% of the weight of SCR[28]. Based on the manufacturers' opinion, while operating with low sulfur fuels, the catalysts of SCR shall be replaced once in a lifetime of the ship.

Table S5: The material and energy details of the marine engine considered in the study.

Material required	Quantity	Reference
Chromium steel (weight ratio %)	40	[25]
Cast iron (weight ratio %)	46	[25]
Aluminum (weight ratio %)	8	[25]
Copper and Zinc (weight ratio %)	0.2	[25]
Lead (weight ratio %)	0.1	[25]
Plastic (weight ratio %)	0.9	[25]
Rubber (weight ratio %)	0.9	[25]
Electricity (kWh/kW)	10.8	[29]

PEMFC: Fuel cells convert the chemical energy of H_2 to electricity through electrochemical oxidation. As the conversion is direct and no need for high combustion temperatures, fuel cells offer higher energy conversion efficiency than conventional energy systems[9]. Low-temperature polymer electrolyte membrane fuel cell (PEMFC) systems are currently the most preferred technology for most transport applications since they have a high power density and allow fast cold start-up and load transient, especially if pure H_2 is available [30]. The materials for PEMFC cell and balance of plant (BoP) are adopted from the study by Stropnik et al. [31] is shown in Table S6.

Table S6:The material and energy details of the PEMFC considered in the study.

Component	Material type	Quantity
PEMFC cell	Graphite (kg/MW)	4 500
	Polyvinyldene chloride (kg/MW)	1 100.
	Aluminum (kg/MW)	300
	Chromium steel (kg/MW)	100
	Glass fibers (kg/MW)	100
	Perfluorosulphonic acid (kg/MW)	70
	Carbon black (kg/MW)	0.8
	Platinum (kg/MW)	0.75
Balance of Plant	Steel product (kg/MW)	3 700
	Polyethylene high density granulate (kg/MW)	1 500
	Chromium steel (kg/MW)	1 100
	Cast iron component (kg/MW)	800
	Aluminum (kg/MW)	750
	Polypropylenes granulate (kg/MW)	250
System	Electricity consumption (kWh/MW)	16 900

SOFC: While using NH_3 as the source of H_2 in a fuel cell, PEMFC is not preferred as even traces of NH3 can poison the fuel cell [30]. The study by Kishimoto et al. [9] demonstrated the performance and durability of NH_3-fueled SOFC stacks. The materials for SOFC cells are still under continuous development, especially for NH_3, the material composition is modeled based on the available literature. For this study, the material composition and weight for the SOFC are based on the study by Al-Khori et al [32] and input obtained from the SOFC manufacturer during the interview. The material for manufacturing is modeled according to the raw material used in the chemical composition and is shown in Table S7, and due to lack of data on the auxiliary materials like solvent, binders, and energy required for producing the stack is not included in analysis.

Table S7:The material and energy details of the SOFC considered in the study.

Component	Material type [9, 32]	Quantity [32]

**SOFC cell**	Cobalt oxide (kg/MW)	46
	Strontium (kg/MW)	7
	Lanthanum (kg/MW)	45
	Nickel oxide (kg/MW)	1 136
	Yttrium oxide (kg/MW)	53
	Zirconia (kg/MW)	358
	Electricity consumption (MJ/MW)	27 280
**Balance of Plant**	Chromium steel (kg/MW)	39 000
	Electricity consumption (MJ/MW)	1 075 000

OCC: Carbon capture is a complex and emerging process currently being implemented now on various industries eg: Boundary Dam Integrated Carbon Capture and Sequestration Demonstration Project in Canada, Gorgon Carbon Dioxide Injection Project in Australia, FutureGen 2.0 Project in the US, etc [33]. Due to limitations such as power demand, volume constraint, and stability, this method is difficult to be installed on ships [33]. There is a wide range of CO2 capture technologies available, including adsorption, cryogenics, absorption, microbial, and membrane [34]. Carbon capture using the chemical solvent Monoethanolamine (MEA) is a proven absorption technology used in various projects [35].

Since the OCC application requires additional power, additional engine power is provided for the operation of CCS making sure that the energy requirement for ship operation is not affected. The CCS capacity is modeled based on previous studies by Luo and Wang [35] and Giordano et al. [36]. The capacity of the CCS system depends on the CO_2 content of exhaust gas is different based on the fuel and the CO_2 capture rate. The OCC system is sized in this study by proportionating the power demand of the CCS system used in the study by Giordano et al. [36]. Based on this the OCC requires 1.4 MW_{el} for capturing CO_2 from the MeOH engine exhaust. It is assumed that the excess heat from the engine can meet the thermal energy requirement for the OCC for all the systems. Kleijn et al. [37] estimated the steel demand for CC installation for 400 MW_{el} is around 3700 tonnes of carbon steel and 950 tonnes of stainless steel. For this study, the materials required are scaled proportionally based on the max CO_2 emission rate from the engine which is 12.73 tCO_2/h, in addition, the compressor capacity of 821 kW is required to liquefy CO_2. The LCI data for compressor is taken from ecoinvent 3.7.1. Due to space limitation of the OCC equipments onboard, the base capture rate considered is 70%, however, for the Monte-Carlo simulation, the capture rate of a minimum of 60% and a maximum of 90% is analyzed.

Reformers: MeOH and NH_3 reformers are used for producing H_2 by cracking the MeOH and NH_3 respectively. The MeOH reformer is used in case 4 and the technology is developed within the HyMethShip project, and it uses the excess heat from the engine for an endothermic reaction between MeOH and water to produce H_2 and CO_2. This reformer has an inbuilt membrane to separate H_2 and CO_2 and thus act as pre-combustion carbon capture. H_2 is directed towards the engine as fuel and CO_2 is liquefied and stored onboard with help of the adsorption chiller. The reactor is having 3 main parts, i) catalyst which supports the cracking process, ii) membrane with sealings for separating H_2 and CO_2, iii) reactor which forms the structure of the reformer. Based on expert feedback, it is assumed that the higher selectivity of the membrane will allow capturing 95% of CO_2 produced and a maximum of 98%. The present result shows that more than 90% capture rate. This range is used in uncertainty analysis (main article Table 5). For case 6, the NH_3 reformer is required only to generate pilot fuel (10% H_2 by volume), and as the by-product is only nitrogen and can be directly used in the engine. However, the temperature for decomposing the

H_2 is between 300 and 520°C depending on the catalyst [30]. Also, the catalyst should be stable in an NH_3 environment at these high temperatures. The detail of the reformer considered for this study is shown in Table S8.

Table S8:The material and energy details of the reformers considered in the study.

Manufacturing data	Methanol reformer	Ammonia reformer
Alumina oxide (kg)	7500	225
Copper oxide (kg)	18000	900
Zinc oxide (kg)	7500	375
Stainless steel (kg)	267000	13350

Absorption chiller system: The absorption chiller system is used to cool down and condense the gaseous CO_2 to liquid CO_2. The absorption chiller system is driven by the excess heat from the main engines. From the manufacturer, the weight of the chiller is calculated for the HyMethShip concept and the major material used in the absorption chiller is stainless steel.

Batteries: Presently, lithium-ion batteries are the most predominant power source for electric vehicles, among which lithium-rich NMC batteries have a higher economical energy density [38]. For this study lithium-ion battery with NMC 811 cathode and a graphite anode is considered. The material data is adopted from the LCA study on a Giga factory for battery manufacturing Chordia et al [39]. Considering the developments of the battery uncertainty analysis is performed for the storage capacity and battery cost which is shown in the main article Table 5.

Motor: The motor is used for electric propulsion and considers a synchronous motor, the weight details are adopted from the manufacturing catalog [40]. The material data and background inventory data are taken from the ecoinvent 3.7.1.

Alternator: The alternator is used for generating electricity onboard from the engines, the power capacity of the alternator depends on the electrical load. The weight details are calculated from the manufacturing catalog [41], and the material composition of the electrical generator is taken from the GREET database [42].

Heat pump: A heat pump is considered for supplying space heat requirements. The material specification and weight are calculated from the manufacturing catalog and material composition is taken from the study by Greening et al. [43] as shown in Table S9.

Table S9: Materials required for the heat pump considered in the study.

Material	Weight (%)
Alloy steel (kg)	15,5%
Reinforcing steel (kg)	58,1%
Copper (kg)	17,7%
Elastomere (kg)	7,7%
polyvinylchloride (kg)	0,8%
HDPE (kg)	0,2%

S2.4 Operation phase

In this phase, emissions from the engine primarily depend on the type of fuel and fuel consumption. Fuel consumption varies with the engine load and for this study, it is assumed that, an average engine load of 80% during cruising and 20% during maneuvering. The fourth IMO GHG study 2020 gives an empirical equation for the specific fuel consumption (SFC) as a function of engine load as shown in equation 1 [44] which is used in this study. The emission inventory for

the cruising and maneuvering is calculated separately. It may be noted that this equation is not applicable for fuel cells or batteries as it operates in modules and load can be varied by not operating some modules [30]. The efficiency of fuel cells decreases at a high load (higher than 80%) as stack voltage decreases due to higher fuel utilization [9]. However, this reduction is not considered in the study as it is assumed that the highest average load is assumed during cruising is 80%. One exemption in the inventory is the emissions considered during the operation phase. Considering that most of the emissions from the propulsion system are released as exhaust gases [6], inventory data during operation does not include emissions to water, soil, and noise.

$$SFC_{ME,load} = SFC_{base} * (0.455 * load^2 - 0.710 * load + 1.280) \quad (1)$$

Since, the engines considered in this study are in different stages of development, from a diesel engine that has been on the market for decades to an H_2 and NH_3 engine that is yet to be tested for marine application. The emissions inventory data and fuel consumption for engines based on fuels MGO and eMeOH (cases 1 to 6) are based on different studies [44-47]. For H_2-fueled engines (cases 7 and 8), the emissions are directly-measured from the engine developed for the HyMethShip project. Fuel consumption for these cases is assumed considering expert opinion. For NH3 engine, the emission of nitrogen compounds is assumed to be the same as MGO and MeOH engines considering that SCR would reduce these emissions with help of a reducing agent. However, less Urea is required for MeOH as the NOx emissions would be less based on the data from the study by Brynolf et al. [47], and based on experts the NH_3 engine doesn't need an external reducing agent as unburned NH_3 act can as the reducing agent. For SCR cases, the NOx and NH_3 emissions are adopted from the study by Andersson et al. [45]. The efficiency of engines is considered based on the experts from the ship design and higher efficiency is considered for CI and DF engines compared to SI engines, also an uncertainty range is considered in the base efficiency of the engine which is shown in the main article Table 5.

For marine applications, NH_3 engines are under development. Engine manufacturer Wärtsilä is presently testing a four-stroke engine running on an NH_3 blend and anticipates the development of the engine on pure NH_3 by the year 2023 [48]. MAN Energy Solutions is also developing an NH_3 two-stroke engine and is anticipated to be ready by 2024 [49]. There are no emission inventory data for this type of engine. In a recent study by Lhuillier et al [12], the NH_3-H_2 mixtures are investigated in an SI engine in a series of experiments and mentioned that higher efficiency was obtained while burning NH_3 with 10% H_2 (by volume) under lean conditions. The results are similar to an early study by Frigo et al.[50]. Considering the poor combustion properties of NH_3, it is assumed that 10% of H_2 by volume is used as a promoter and it is produced from NH_3 with help of a reformer. Based on 'experts' opinion, the emission-related to carbon would be the same as an H_2 engine assuming that this is not related to the burning of fuels as there are no carbon atoms in the fuel. These studies suggest that the exhaust contains high unburned NH_3 and NOx emissions because of fuel-bound nitrogen [12, 50]. The SCR can be used for converting the NOx using NH_3 in the exhaust. The experts suggest that proper tuning of the engine for optimal combustion of NH_3 can ensure that the NH_3 slip is enough to reduce NOx in SCR and can meet tier III requirements. Based on this it is assumed that the NH_3 and NOx emissions after SCR would be similar to emissions from current SCR systems. However, the emission of nitrous oxide is uncertain and for this study an uncertainty analysis is done with emission from 0.013 to 0.13 g/kWh (10 times) to understand the impact on GWP.

For PEMFC, during the electrochemical reaction, only water is produced as a byproduct and for SOFC, nitrogen and water are byproducts of combustion and based on experts, there won't be any notable emission of pollutants from fuel cells. The SOFC operates at high temperatures and the heat from exhaust can be used for space heating. The efficiency of different components also affects fuel consumption. Following are the assumptions considered in the study efficiency of 98% for electric motor [51], efficiency of 97% for alternator [41], 98% efficiency for the gearbox [51], 98% efficiency for the control unit [26], and COP of 4 for the heat pump [52].

Inventory related to the replacement, for optimal case the catalyst in the reformer, should be replaced every 3 years averaging to 7 times during 25 years of lifetime. The Batteries and fuel cells are considered to be replaced twice during 25 years of lifetime. It may be noted that the battery would either discharge during maneuvering and cruising or charge during mooring making it in active operation the entire period. Whereas fuel cells would be idle during the mooring. SCR catalyst is considered to be replaced once during the ship's lifetime.

For the HyMethShip system de-ionized water is required in the reformer (0.33 kg H_2O for 1 kg H_2). For PostCC operation, due to the degradation of the solvent, there are some materials that to be supplemented and some related emissions to air [36] as shown in Table S10.

Table S10: Consumables required for PostCC operation and emissions during the operation.

**Consumables *required***	***Quantity***
MEA,(kg/tCO$_2$in)	1.44
Caustic soda (kg/tCO$_2$in)	0.12
Activated carbon, (kg/tCO$_2$in)	0.07
Water (kg/tCO$_2$in)	18.1
Air emissions	***Quantity***
Ar, (kg/tCO$_2$in)	54.8
N2, (kg/tCO$_2$in)	3202.2
O2, (kg/tCO$_2$in)	128.3
MEA, (kg/tCO$_2$in)	0.06
NH$_3$, (kg/tCO$_2$in)	0.03
Formaldehyde, (kg/tCO$_2$in)	2.4×10^{-4}
Acetaldehyde, (kg/tCO$_2$in)	1.5×10^{-4}

S2.5 Cost details

Total life cycle cost includes acquisition cost (C_A), fuel cost (C_F), operation and maintenance cost (C_{OMC}), replacement cost (C_R), external cost (C_E), EOL cost (C_{EOL}), and revenue loss (C_{RL}). C_A, C_R, and maintenance and operation part of C_{OMC} are based on Table 3 in the article. The consumable part of C_{OMC} cost includes urea consumption based on Table 4 and other consumptions like MEA, de-ionized water, caustic soda, etc. based on data given in S2.4. The unit price of the consumable is taken from the current market price. C_{EOL} is the end of life cost is calculated based on the recycling value of the material as shown in Table S11. C_{RL} is calculated using additional volume occupied by the different propulsion systems including the storage tanks, whose value is shown in Table S12. Whereas C_E is included in sensitivity analysis considering carbon tax for fossil-based emissions,

Table S11: The scrap cost of the material during the end of life.

Material	*Value (€/tonne)*	*Reference*
aluminium	1680	[53]
cast iron	126	[53]
chromium	600	[54]
lead	1512	[54]
nickel	5278	[55]
steel, chromium steel 18/8	898,8	[54]
titanium dioxide	2000	[54]
copper	4368	[54]
zinc	672	[54]
Battery	2407	[55]
platinum	14815	[55]

Table S12: The volume and weight of the propulsion system for the cases considered in the study.

	Case 1 eMeOHICE	Case 2 eMeOHICE w PostCC	Case 3 HyMethShip	Case 4 eLH2ICE	Case 5 eNH3ICE	Case 6 eLH2PEMFC	Case 7 eNH3SOFC	Case 8 BE	Case 9 MGO ICE
Volume (m^3)	764	1501	1383	1333	1129	798	1064	1743	598
Weight (tonnes)	603	1178	1121	775	875	661	1010	3554	501

Fuel distribution and storage cost is not included in the main analysis, however, the impact of this cost on total LCC is analyzed using the data listed in Table S13.

Table S13: Fuel distribution cost used for the study

Fuel	*Cost(€/GJ)*
Electricity [56]	10.2c
Liquified hydrogen (LH2) [57]	11.6
Methanol [57]	0.6
Ammonia [57]	1.2
Fossil conventional alternative [57]	0.2

S2.6 Datasets

Different raw materials are used for manufacturing the components, and the LCI detail for these materials are taken from the existing database as a background process (Table S14).

Table S14:Materials considered in the manufacturing of different components and relation to the database.

Materials	Used in	Database	Description in database
		Plastics	
Polyethylene	Heat pump, LTPEMFC	Ecoinvent 3.7.1	market for polyethylene, high density, granulate | polyethylene, high density, granulate | Cutoff, S - GLO
Polyvinyl chloride	Heat pump, LTPEMFC	Ecoinvent 3.7.1	market for polyvinylchloride, suspension polymerised | polyvinylchloride, suspension polymerised | Cutoff, S - GLO
Nylon	Marine engine	Ecoinvent 3.7.1	market for nylon 6 | nylon 6 | Cutoff, S - RER
Silicon	Marine engine	Ecoinvent 3.7.1	market for silicone product | silicone product | Cutoff, S - RER
Nafion	LTPEMFC	Ecoinvent 3.7.1	Nafion
Polypropylene	LTPEMFC	Ecoinvent 3.7.1	market for polypropylene, granulate | polypropylene, granulate | Cutoff, S - GLO
Glass fibre	LTPEMFC	Ecoinvent 3.7.1	market for glass fibre | glass fibre | Cutoff, S - GLO
		Metals	
Chromium steel	Heat pump, SCR, OCC, alternator, marine engine, CO2 tank, gearbox, LTPEMFC, SOFC	Ecoinvent 3.7.1	market for steel, chromium steel 18/8 | steel, chromium steel 18/8 | Cutoff, S - GLO
Nickel	Heat pump, SOFC, Alkaline electrolyzers	Ecoinvent 3.7.1	market for nickel, class 1 | nickel, class 1 | Cutoff, S – GLO
Steel	Alkaline electrolyzer, LTPEMFC	Ecoinvent 3.7.1	market for steel, unalloyed | steel, unalloyed | Cutoff, S – GLO
Lead	Heat pump, marine engine	Ecoinvent 3.7.1	market for lead | lead | Cutoff, S – GLO
Zinc	Marine engine	Ecoinvent 3.7.1	market for zinc | zinc | Cutoff, S - GLO
Cast iron	Marine engine, OCC, LTPEMFC	Ecoinvent 3.7.1	market for cast iron | cast iron | Cutoff, S - GLO
Copper	Heat pump, alternator, marine engine	Ecoinvent 3.7.1	market for wire drawing, copper | wire drawing, copper | Cutoff, S - GLO
Aluminium	Heat pump, alternator, marine engine, LTPEMFC	Ecoinvent 3.7.1	market for aluminium, primary, ingot | aluminium, primary, ingot | Cutoff, S - IAI Area, EU27 & EFTA
Platinum	LTPEMFC	Ecoinvent 3.7.1	market for platinum | platinum | Cutoff, S - GLO
Lanthanum	SOFC	Ecoinvent 3.7.1	market for lanthanum oxide | lanthanum oxide | Cutoff, S - GLO
Zirconia	SOFC	Ecoinvent 3.7.1	market for zirconium oxide | zirconium oxide | Cutoff, S - GLO
Yttria	SOFC	Ecoinvent 3.7.1	market for yttrium oxide | yttrium oxide | Cutoff, S - GLO
		Mineral and Ceramics	
Titanium dioxide	SCR	Ecoinvent 3.7.1	market for titanium dioxide | titanium dioxide | Cutoff, S - RER
Zinc oxide	Methanol reformer, ammonia reformer	Ecoinvent 3.7.1	market for zinc oxide | zinc oxide | Cutoff, S - GLO
copper oxide	Methanol reformer, ammonia reformer	Ecoinvent 3.7.1	Copper oxide production | copper oxide | Cutoff, S - GLO
Alumina	Methanol reformer, ammonia reformer	Ecoinvent 3.7.1	market for aluminium oxide, metallurgical | aluminium oxide, metallurgical | Cutoff, EU27 & EFTA
cobalt oxide	SOFC	Ecoinvent 3.7.1	market for cobalt oxide | cobalt oxide | Cutoff, S - GLO
		Other materials	
De-ionised water	Electrolysis, Reformer operation, OCC operation	Ecoinvent 3.7.1	market for water, deionised | water, deionised | Cutoff, S - Europe without Switzerland
MEA	OCC operation, DAC operation	Ecoinvent 3.7.1	market for monoethanolamine
Sodium hydroxide	OCC operation	Ecoinvent 3.7.1	market for sodium hydroxide, without water, in 50% solution state | sodium hydroxide, without water, in 50% solution state | Cutoff, S - GLO
Urea	SCR operation	Ecoinvent 3.7.1	market for urea | urea | Cutoff, S - RER
Potassium hydroxide	Alkaline electrolysis	Ecoinvent 3.7.1	potassium hydroxide production | potassium hydroxide | Cutoff, S
Carbon black	LTPEMFC	Ecoinvent 3.7.1	market for carbon black | carbon black | Cutoff, S - GLO
Graphite	LTPEMFC	Ecoinvent 3.7.1	market for anode, graphite, for lithium-ion battery | anode, graphite, for lithium-ion battery | Cutoff, S - GLO

S3: Life cycle assessment result

S3.1 Environmental impact assessment

The pLCA results for different impact categories (other than GWP) without normalization at various life cycle phases are shown in Figures S4 to S12. Impact categories are assessed according to Environmental Footprint (EF) 3.0 LCIA method recommended by the European Commission's Joint Research Centre.

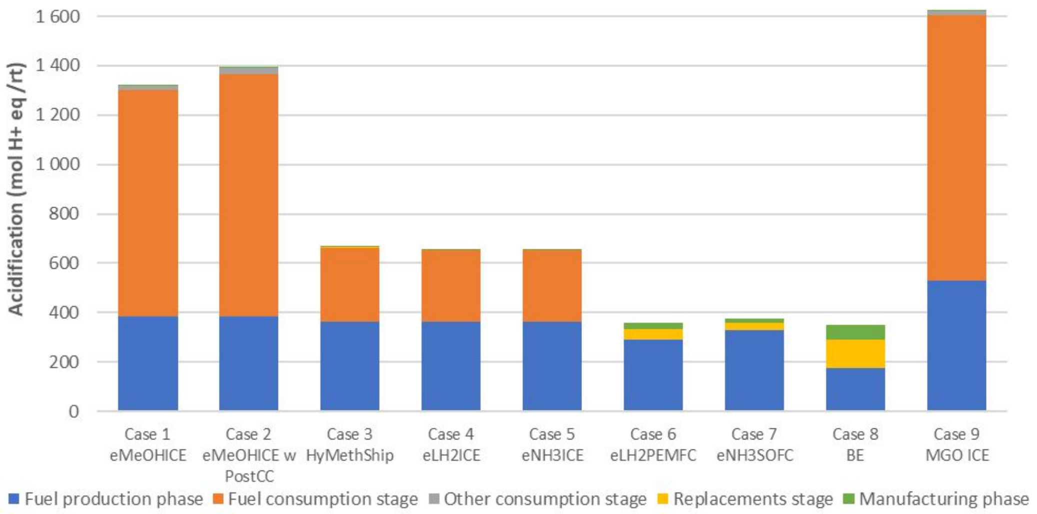

Figure S5:pLCA results of different propulsion options for impact category acidification for the round trip.

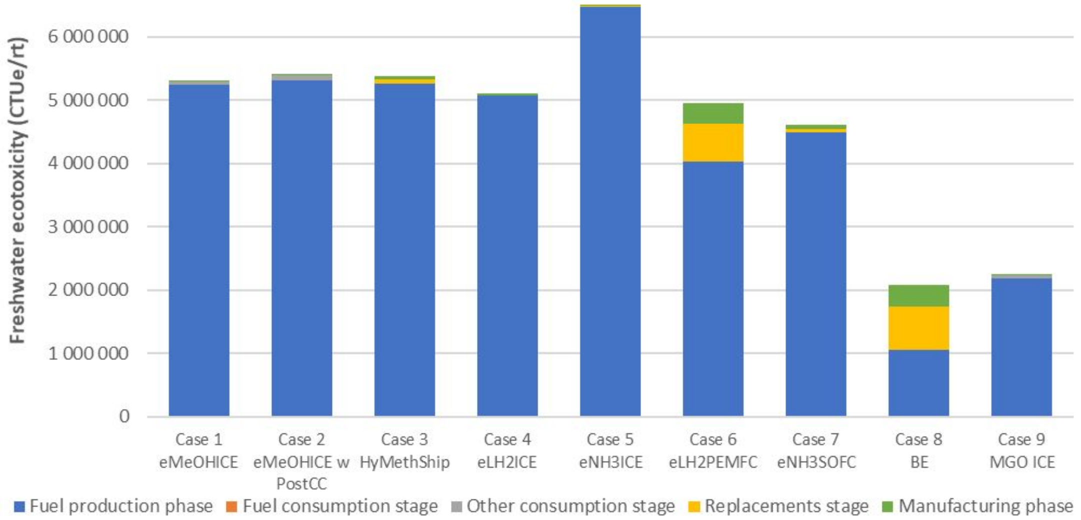

Figure S6:pLCA results of different propulsion options for impact category freshwater ecotoxicity for the round trip.

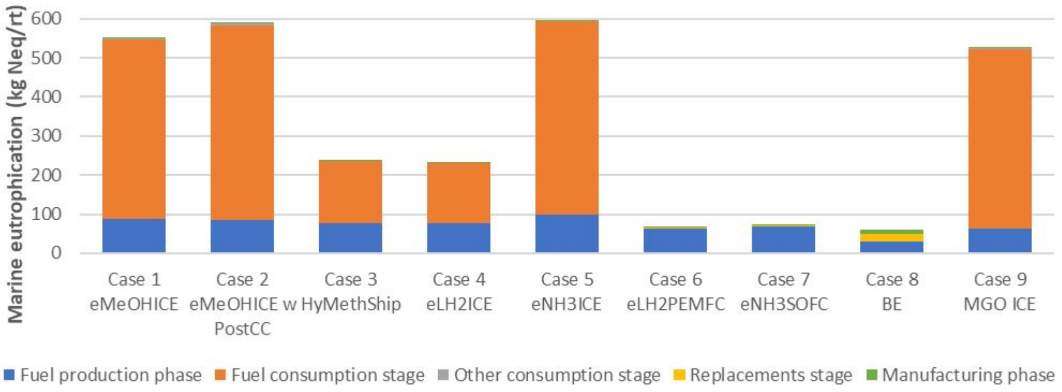

Figure S7:pLCA results of different propulsion options for impact category marine eutrophication for the round trip.

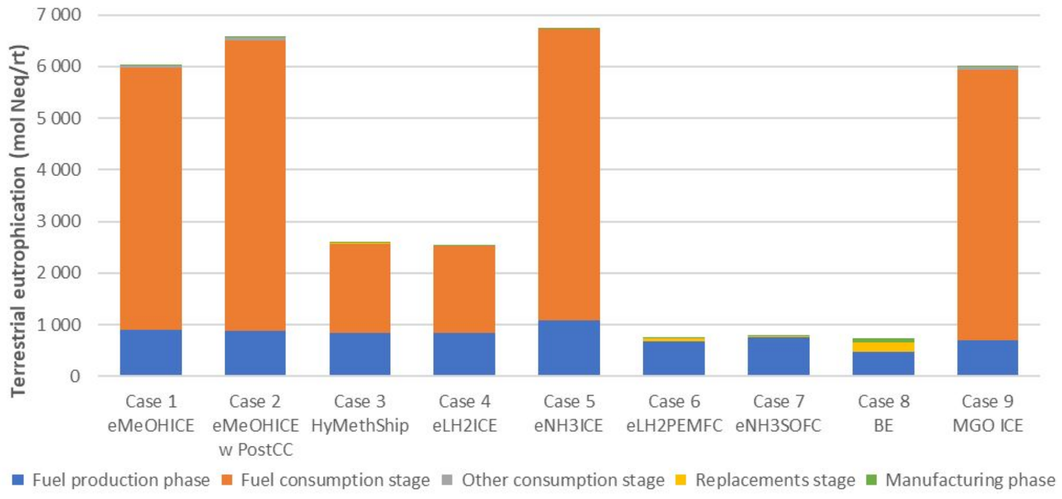

Figure S8:pLCA results of different propulsion options for impact category terrestrial eutrophication for the round trip.

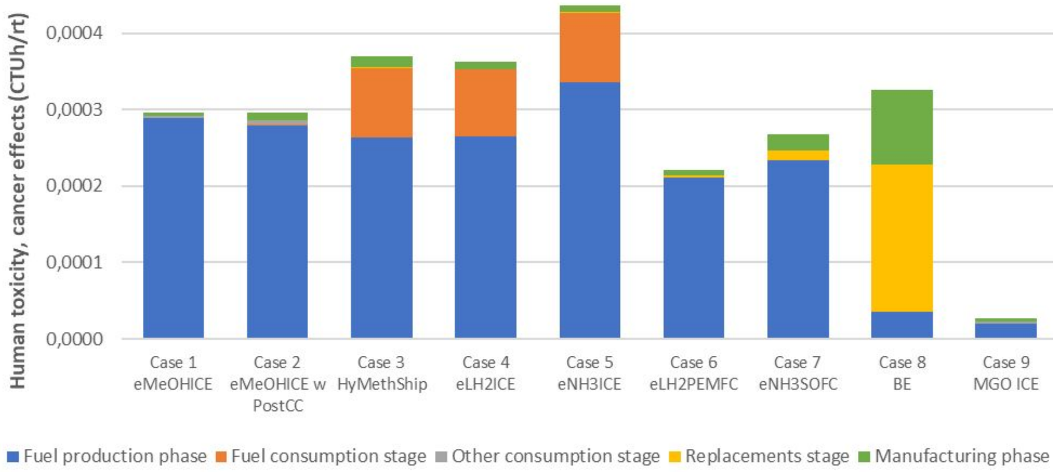

Figure S9:pLCA results of different propulsion options for impact category human toxicity, cancer effects for the round trip.

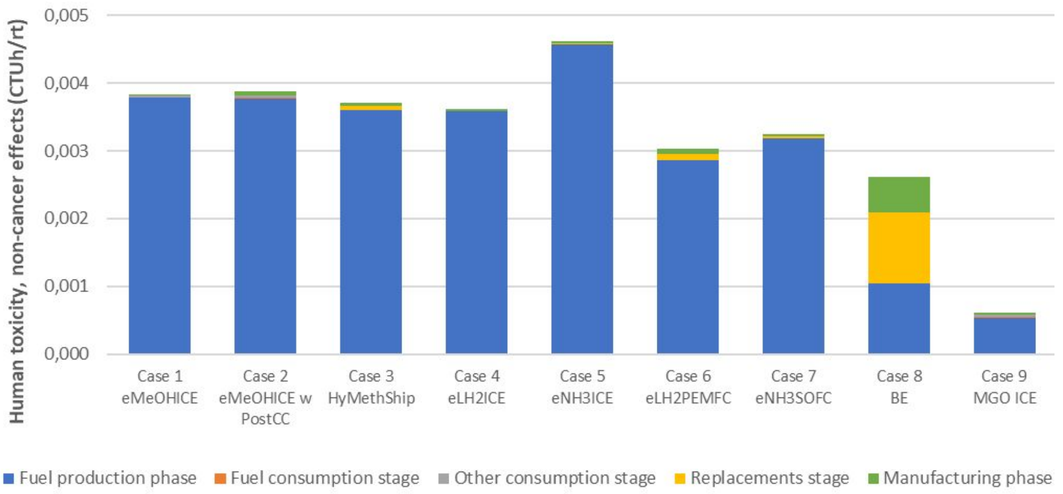

Figure S10: pLCA results of different propulsion options for impact category human toxicity, non-cancer effects for the round trip.

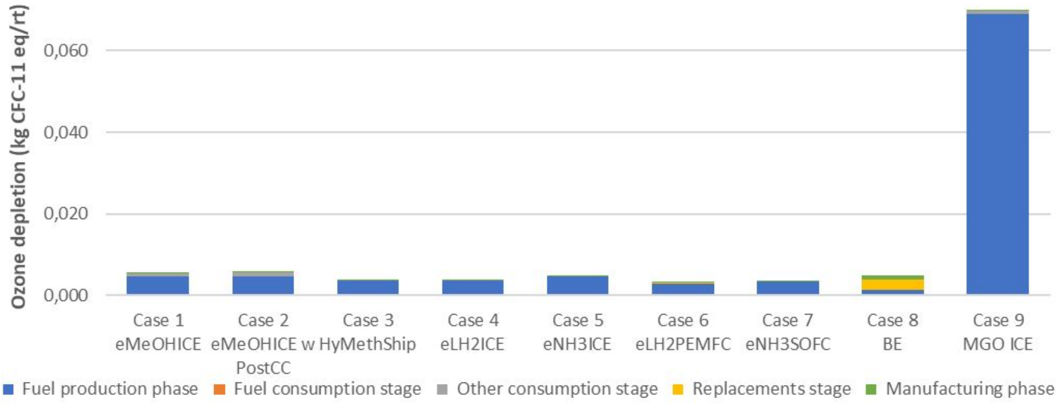

Figure S11: pLCA results of different propulsion options for impact category ozone depletion for the round trip.

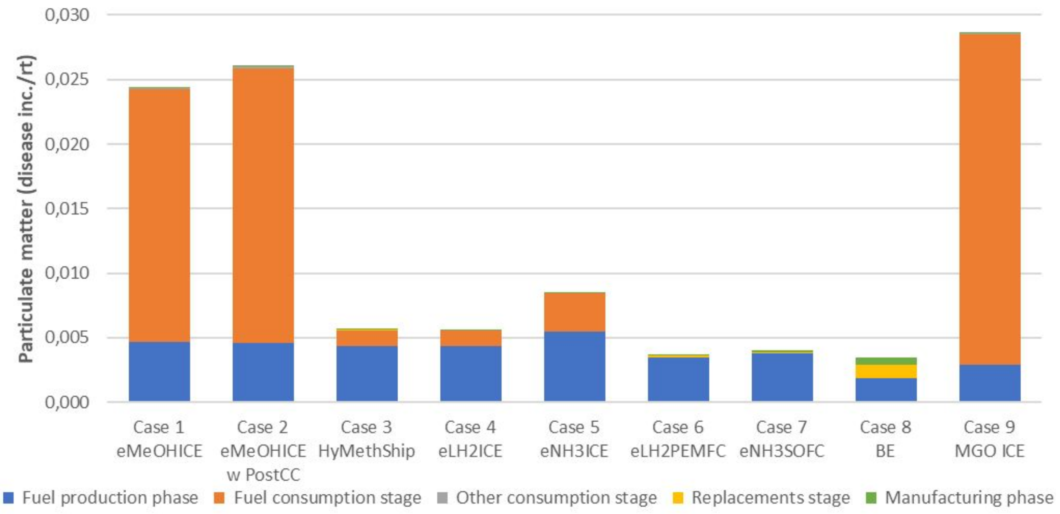

Figure S12: pLCA results of different propulsion options for impact category particulate matter for the round trip.

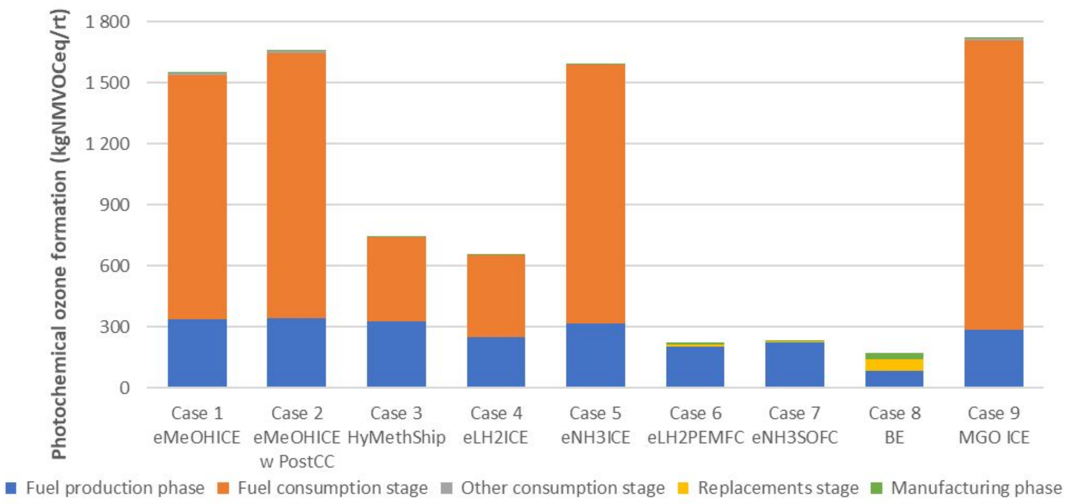

Figure S13: pLCA results of different propulsion options for impact category photochemical ozone formation for the round trip.

S3.2 Scenario analysis

There are several possible configurations while designing and operating a B.E ship, three possibilities as listed in Table 5. Figure S13 shows the climate change impact (GWP20 and GWP 100) for these three scenarios. The main observations are: First, there is a significant difference in the GWP potential with different configurations and is mainly associated with the carbon intensity of electricity used. Second, the lowest GWP is for the configuration where the electricity comes from wind power with the battery storage infrastructure. This indicates that the port facility to store the electricity from renewables is promising for BE. Third, having onboard batteries for the round trip is better than having only for a single trip as it allows charging from cleaner electricity. In the case of b, the GWP is high due to the higher carbon intensity of the German mix. However, additional space and weight are required onboard for round trip considering the lower volumetric and gravimetric energy density of battery packs.

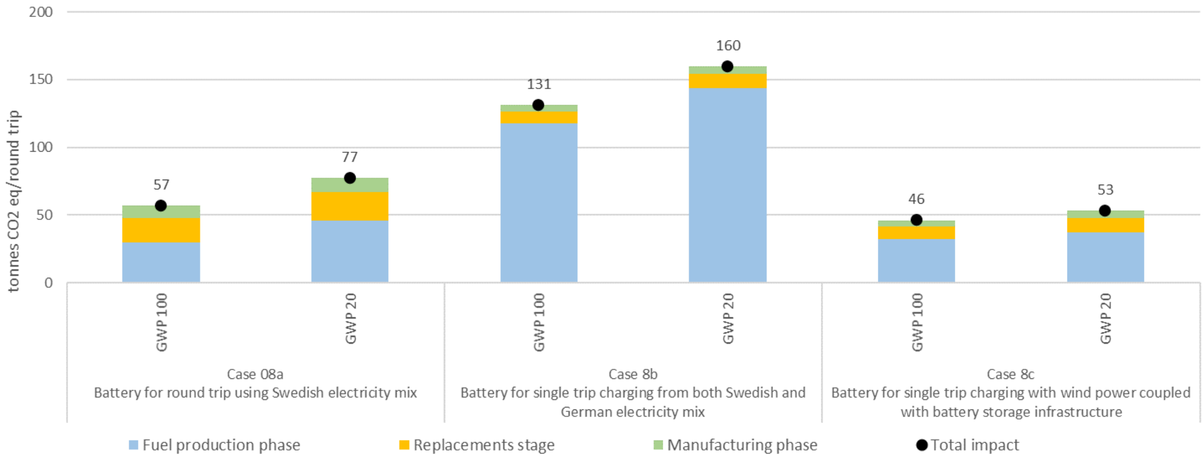

Figure S14: Climate change impact for different scenarios for BE option.

S3.3 Uncertainty analysis

The uncertainty ranges of the result based on the parameters listed in Table 5 of the main article for all the categories are shown in Figures S14 to S24 for the assessed decarbonized pathways. The same methodology is used for the assessment, that is IPCC AR6 for GWP and EF3.0 for other impact categories. The result shows that all options remain effective to reduce the GWPs, however, the uncertainty in the nitrous oxide emission makes eNH3ICE more uncertain.

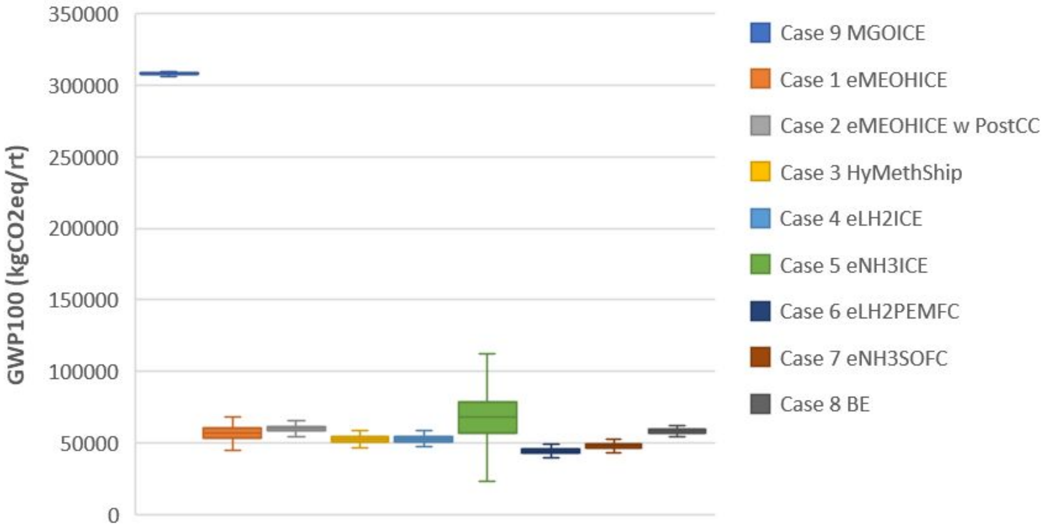

Figure S15:Monte-Carlo simulation results of different propulsion options for impact category climate change GWP100.

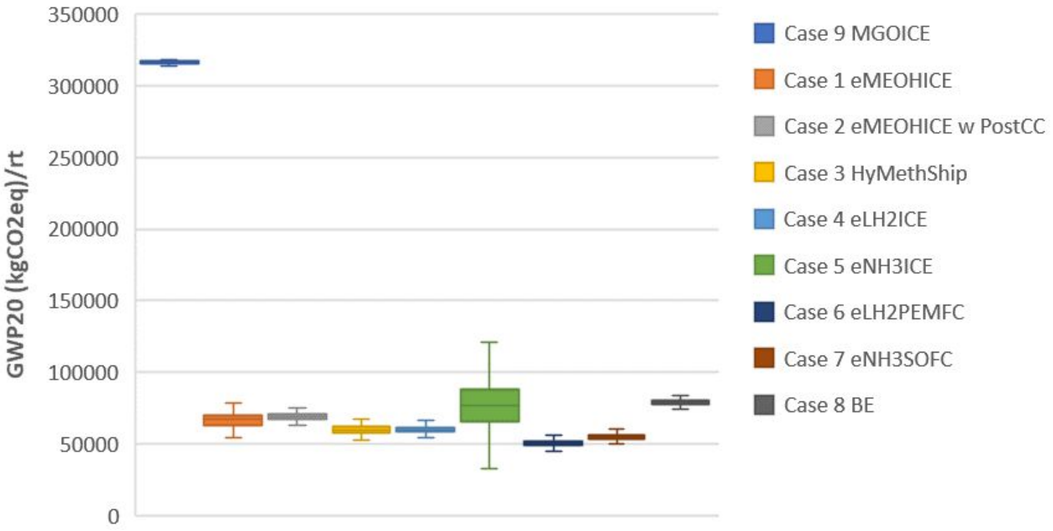

Figure S16:Monte-Carlo simulation results of different propulsion options for impact category climate change GWP20.

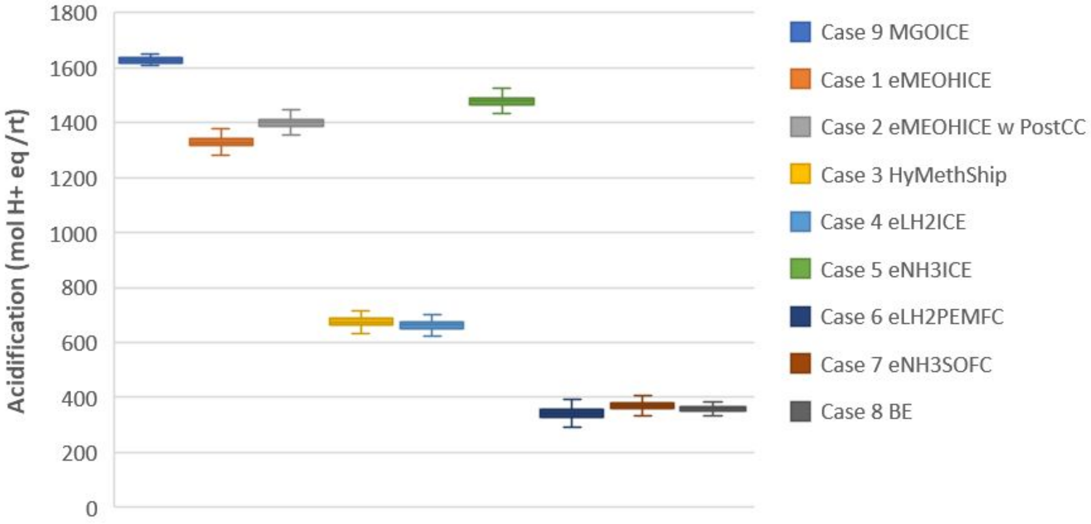

Figure S17:Monte-Carlo simulation results of different propulsion options for impact category acidification for the round trip.

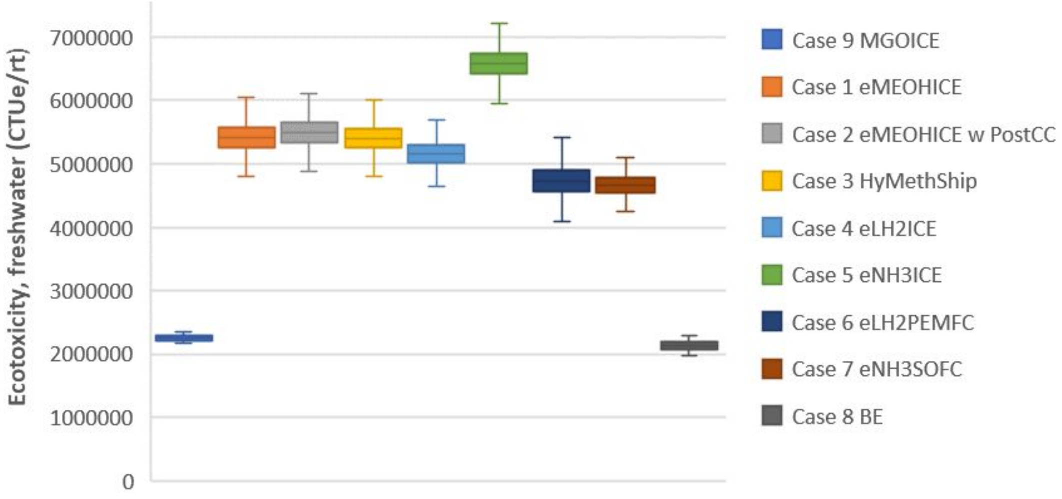

Figure S18:Monte-Carlo simulation results of different propulsion options for impact category freshwater ecotoxicity.

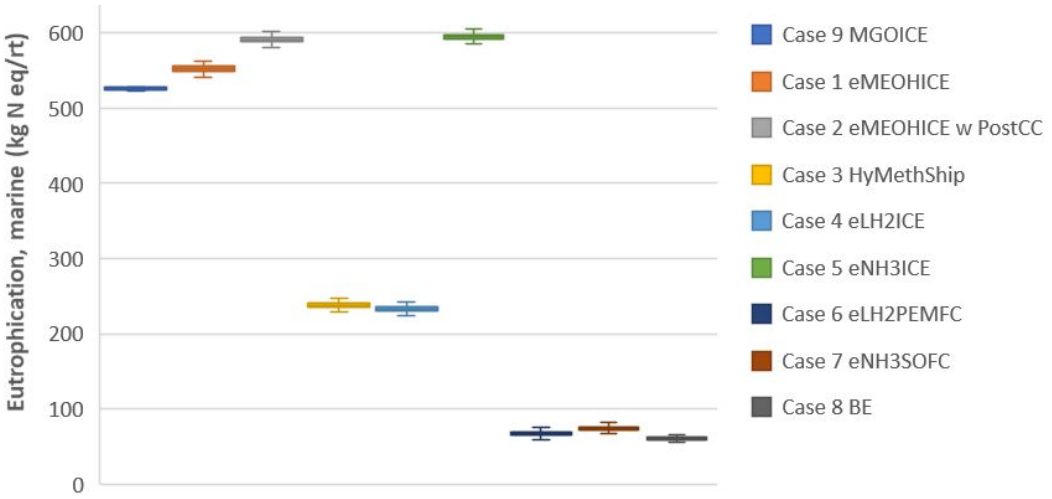

Figure S19: Monte-carlo simulation results of different propulsion options for impact category marine eutrophication.

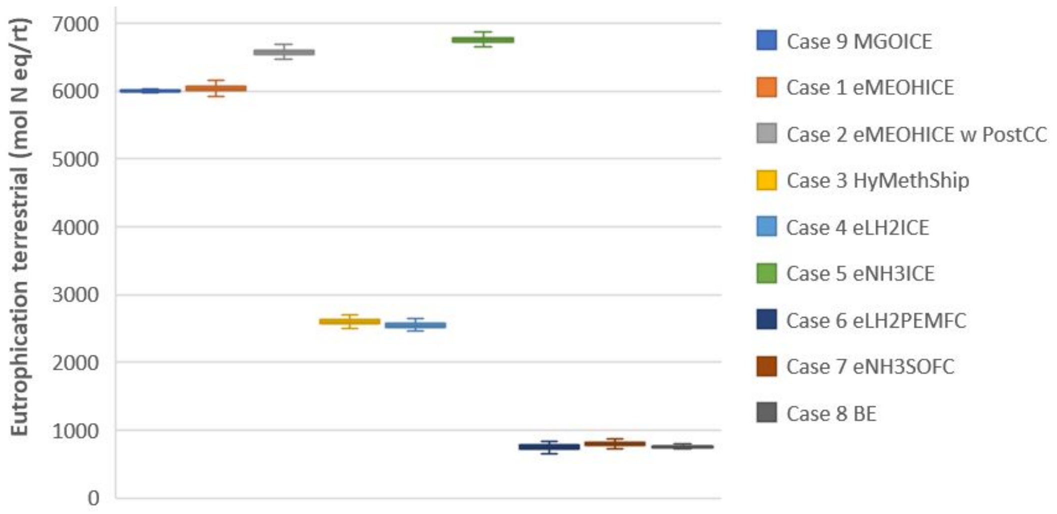

Figure S20:Monte-Carlo simulation results of different propulsion options for impact category eutrophication terrestrial.

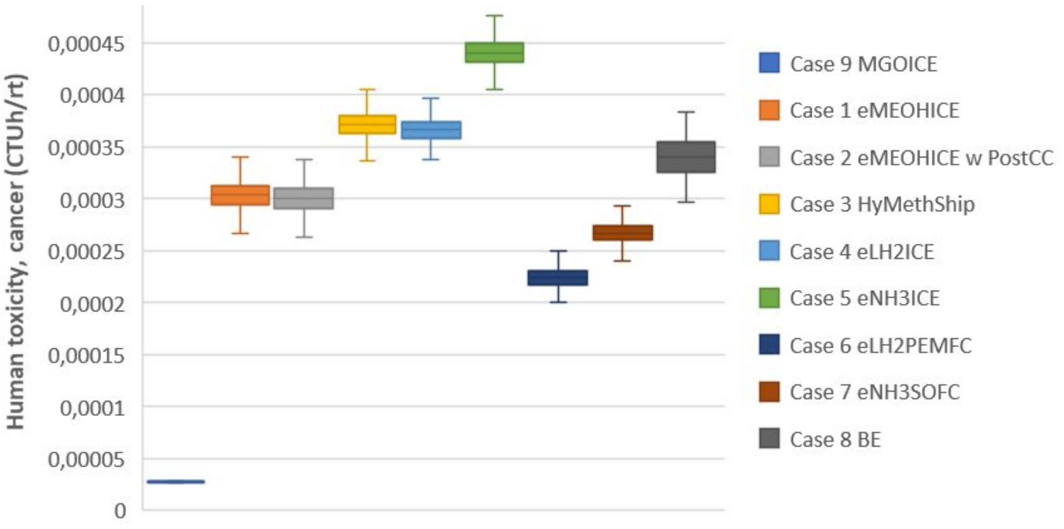

Figure S21:Monte-Carlo simulation results of different propulsion options for impact category human toxicity, cancer effect.

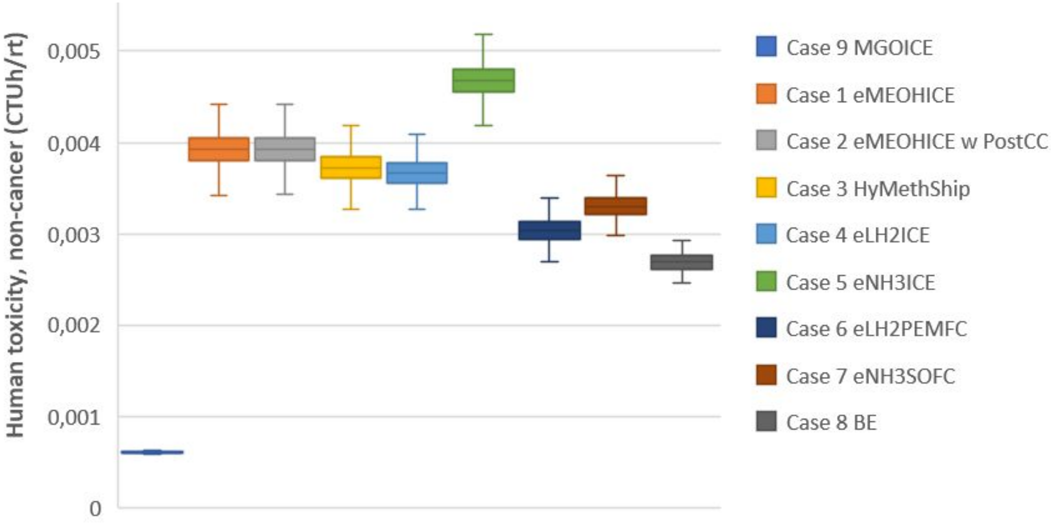

Figure S22:Monte-Carlo simulation results of different propulsion options for impact category human toxicity, non-cancer effect.

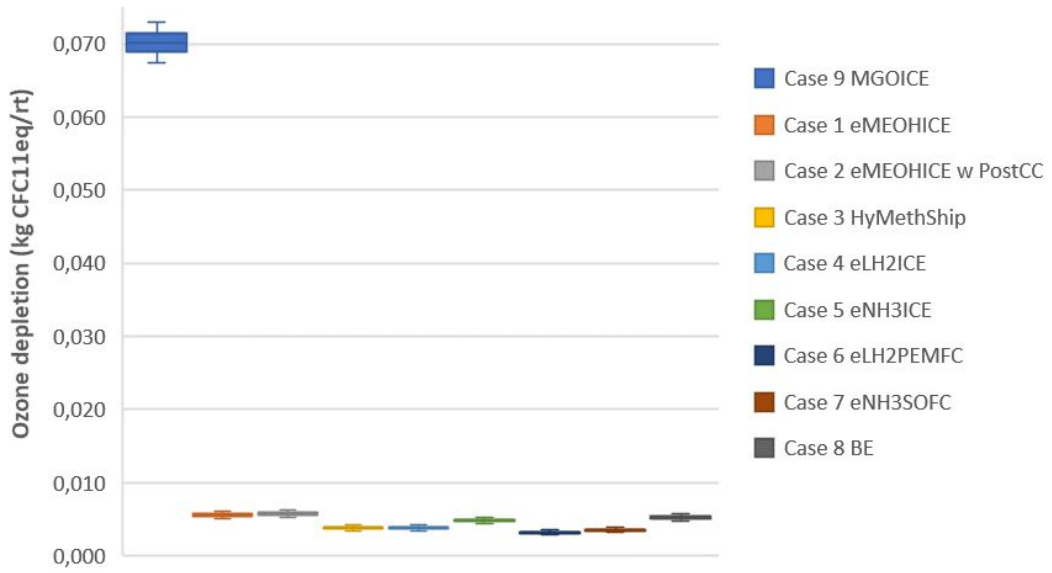

Figure S23: Monte-Carlo simulation results of different propulsion options for impact category ozone depletion.

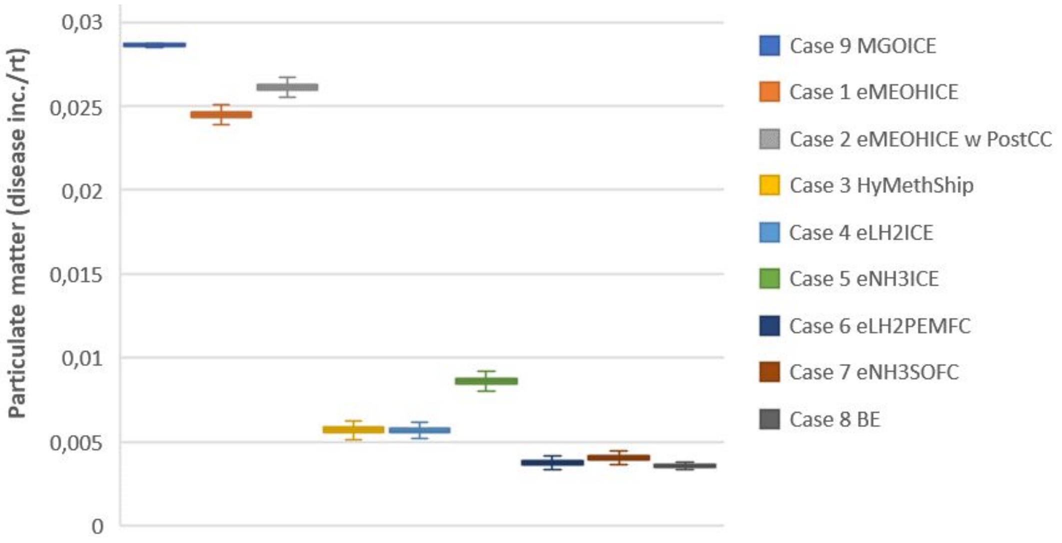

Figure S24:Monte-Carlo simulation results of different propulsion options for impact category particulate matter.

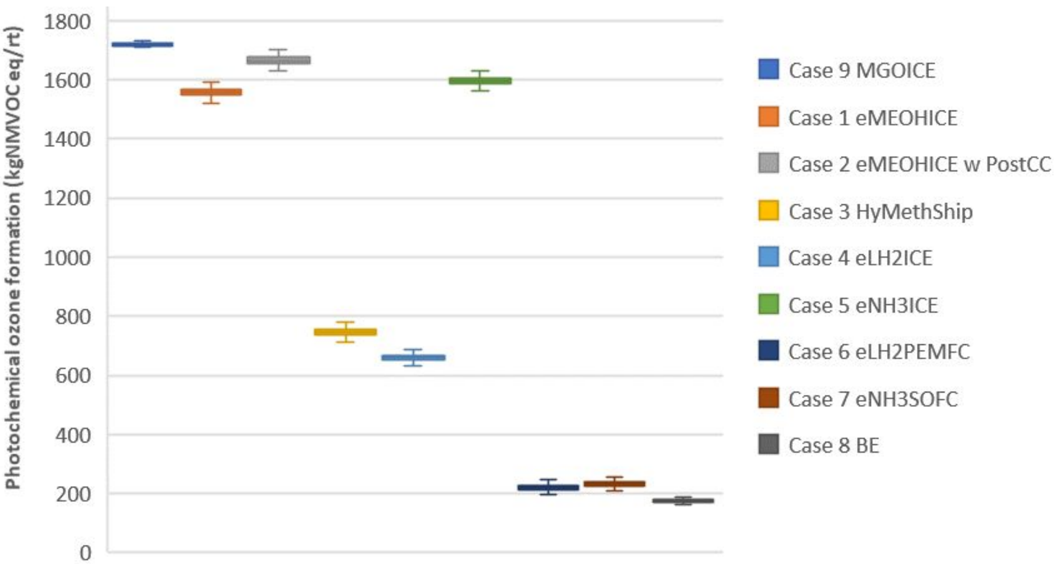

Figure S25:Monte-Carlo simulation results of different propulsion options for impact category photochemical ozone formation.

S4: Life cycle cost results

S4.1 Fuel cost uncertainty

Most of the uncertainties considered in this study are linked to fuel production including the cost of the electricity, the energy required for different processes, the uncertainty in the investment cost, etc. For the eMeOH option, the recirculation of CO_2 captured onboard reduces the MeOH cost as the cost of capturing CO_2 from the DAC is expensive compared to other options. Figure S25 illustrates the result of uncertainty analysis for the cost of fuel.

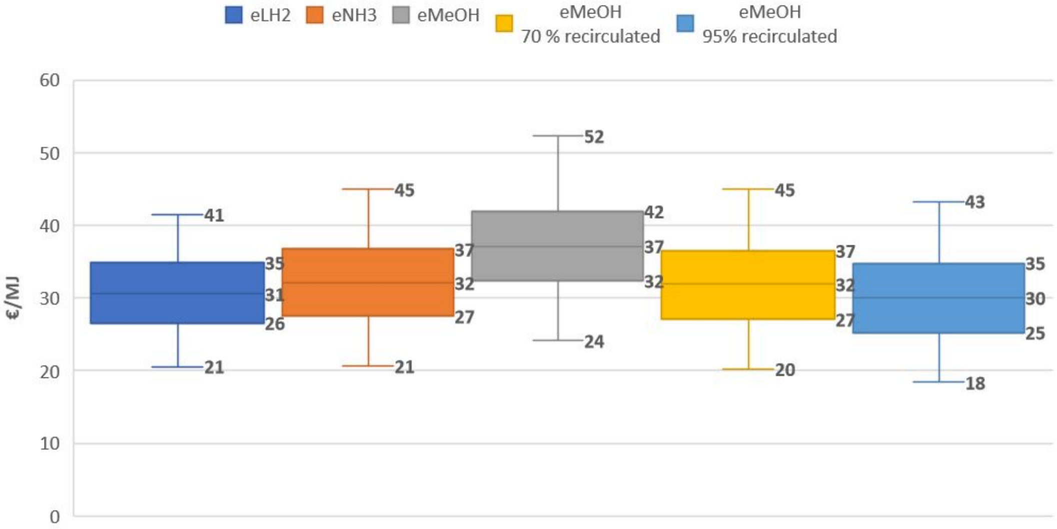

Figure S26:Cost of the fuel calculated and used for the calculation of LCC. The uncertainty range is based on the Monte-Carlo analysis.

S4.2 Uncertainty analysis with port infrastructure

The cost of distribution varies between the fuel, even though it is not added in the main analysis but is analyzed based on data shown in Table S13. Figure S26 illustrates the total eLCC results including fuel distribution cost, and the result shows that the eLH_2 options have the highest impact

of around 30% of the total cost, followed by BE options with 17% of the total cost. These have a significant change in the ranking of the options based on the cost.

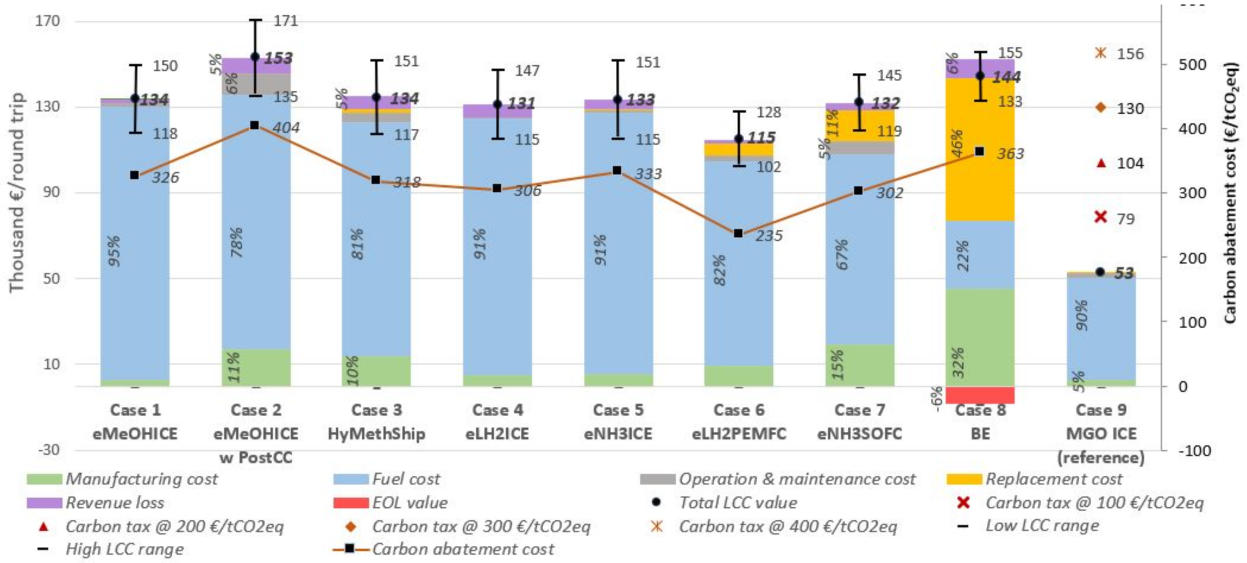

Figure S27:eLCC results including the cost associated without the fuel distribution.

References

[1] *ISO 14044: 2006, Environmental management — Life cycle assessment*, International Organization for Standardization, 2006.

[2] S. Cucurachi, C. van der Giesen, and J. Guinée, "Ex-ante LCA of Emerging Technologies," *Procedia CIRP,* vol. 69, pp. 463-468, 2018, doi: 10.1016/j.procir.2017.11.005.

[3] R. Arvidsson *et al.*, "Environmental Assessment of Emerging Technologies: Recommendations for Prospective LCA," *Journal of Industrial Ecology,* vol. 22, no. 6, pp. 1286-1294, 2018, doi: https://doi.org/10.1111/jiec.12690.

[4] European Commission - Joint Research Centre, "International Reference Life Cycle Data System (ILCD) Handbook - General guide for Life Cycle Assessment - Detailed guidance," Institute for Environment and Sustainability,, Luxembourg, 2010.

[5] J.-M. Rödger, L. L. Kjær, and A. Pagoropoulos, "Life Cycle Costing: An Introduction," in *Life Cycle Assessment*, 2018, ch. Chapter 15, pp. 373-399.

[6] E. Malmgren, S. Brynolf, E. Fridell, M. Grahn, and K. Andersson, "The environmental performance of a fossil-free ship propulsion system with onboard carbon capture – a life cycle assessment of the HyMethShip concept," *Sustainable Energy Fuels,* vol. 5, pp. 2753-2770, 2021, doi: 10.1039/D1SE00105A.

[7] ABS, "Ammonia as marine fuel," October 2020.

[8] K. Kim, G. Roh, W. Kim, and K. Chun, "A Preliminary Study on an Alternative Ship Propulsion System Fueled by Ammonia: Environmental and Economic Assessments," *Journal of Marine Science and Engineering,* vol. 8, no. 3, 2020, doi: 10.3390/jmse8030183.

[9] M. Kishimoto *et al.*, "Development of 1 kW-class Ammonia-fueled Solid Oxide Fuel Cell Stack," *Fuel Cells,* vol. 20, no. 1, pp. 80-88, 2020, doi: 10.1002/fuce.201900131.

[10] Green/EFA, "Towards an emission trading scheme that delivers a green and just transformation of European energy and industry," 2021. [Online]. Available: https://www.greens-efa.eu/en/article/document/towards-an-ets-that-works-for-climate-and-the-transformation-of-industry

[11] A. D. Korberg, S. Brynolf, M. Grahn, and I. R. Skov, "Techno-economic assessment of advanced fuels and propulsion systems in future fossil-free ships," (in English), *Renew Sust Energ Rev,* vol. 142, May 2021, doi: 10.1016/j.rser.2021.110861.

[12] C. Lhuillier, P. Brequigny, F. Contino, and C. Rousselle, "Performance and Emissions of an Ammonia-Fueled SI Engine with Hydrogen Enrichment," presented at the SAE Technical Paper Series, 2019.

[13] A. Valera-Medina, H. Xiao, M. Owen-Jones, W. I. F. David, and P. J. Bowen, "Ammonia for power," *Progress in Energy and Combustion Science,* vol. 69, pp. 63-102, 2018, doi: 10.1016/j.pecs.2018.07.001.

[14] European Commission, "EU Reference Scenario 2020- Energy. transport and GHG emissions trends to 2050," European Commission, Brussels, 2021.

[15] NCE Maritime CleanTech, "Norwegian future value chains for liquid hydrogen," 2019.

[16] J. Q. Mathieu Delpierre, Jan Mertens, Anne Prieur-Vernat, Stefano Cucurachi, "Assessing the environmental impacts of wind-based hydrogen production in the Netherlands using ex-ante LCA and scenarios analysis," *Journal of Cleaner Production,* vol. 299, 2021.

[17] Danish Energy Agency, "Technology data for renewable fuels. Updated July 2020," Copenhagen, 2017.

[18] K. Stolzenburg *et al.*, "Efficient Liquefaction of Hydrogen: Results of the IDEALHY Project," presented at the XXth energie – symposium, Stralsund, Germany, 7 – 9 November, 2013.

[19] A. A. Kiss, J. J. Pragt, H. J. Vos, G. Bargeman, and M. T. de Groot, "Novel efficient process for methanol synthesis by CO 2 hydrogenation," *Chem. Eng. J.,* vol. 284, pp. 260-269, 2016, doi: 10.1016/j.cej.2015.08.101.

[20] S. Deutz and A. Bardow, "Life-cycle assessment of an industrial direct air capture process based on temperature–vacuum swing adsorption," *Nature Energy,* vol. 6, no. 2, pp. 203-213, 2021, doi: 10.1038/s41560-020-00771-9.

[21] C. Smith, A. K. Hill, and L. Torrente-Murciano, "Current and future role of Haber–Bosch ammonia in a carbon-free energy landscape," *Energy & Environmental Science,* vol. 13, no. 2, pp. 331-344, 2020, doi: 10.1039/c9ee02873k.

[22] S. A. Nosherwani and R. C. Neto, "Techno-economic assessment of commercial ammonia synthesis methods in coastal areas of Germany," *Journal of Energy Storage,* vol. 34, 2021, doi: 10.1016/j.est.2020.102201.

[23] K. Stolzenburg and R. Mubbala, "Integrated Design for Demonstration of Efficient Liquefaction of Hydrogen (IDEALHY Deliverable 3.16)," Fuel Cells and Hydrogen Joint Undertaking (FCH JU), 16 December 2013.

[24] Danish Energy Agency, "Technology Data – Carbon Capture, Transport and Storage, November 2021," Copenhagen, 2021.

[25] B. Jeong, H. Wang, E. Oguz, and P. Zhou, "An effective framework for life cycle and cost assessment for marine vessels aiming to select optimal propulsion systems," *Journal of Cleaner Production,* vol. 187, pp. 111-130, 2018, doi: 10.1016/j.jclepro.2018.03.184.

[26] *Diesel Electric Propulsion plant*, Man Energy solutions.

[27] R. Parsmo, K. Yaramenka, H. Winnes, and E. Fridell, "NOX Abatement in the Baltic Sea- An Evaluation of Different Policy Instruments," IVL Swedish Environmental Research Institute Ltd., C 247, 2017.

[28] Z. Liang, X. Ma, H. Lin, and Y. Tang, "The energy consumption and environmental impacts of SCR technology in China," *ApEn,* vol. 88, no. 4, pp. 1120-1129, 2011, doi: 10.1016/j.apenergy.2010.10.010.

[29] Q. Jiang, Z. Liu, T. Li, H. Zhang, and A. Iqbal, "Life Cycle Assessment of a Diesel Engine Based on an Integrated Hybrid Inventory Analysis Model," *Procedia CIRP,* vol. 15, pp. 496-501, 2014, doi: 10.1016/j.procir.2014.06.091.
[30] L. v. Biert, "Solid oxide fuel cells for ships-System integration concepts with reforming and thermal cycles," *Technische Universiteit Delft,* 2020, doi: 10.4233/uuid:dd1f7899-38ee-4c78-a5b0-a6fa92c90f56.
[31] R. Stropnik;, M. Sekavčnik;, A. Lorrič;, and M. Mori, "Life Cycle Assessment of 1kW PEMFC system with the focus on critical materials," presented at the 7th International Youth Conference on Energy (IYCE), 3-6 July 2019, 2019
[32] K. Al-Khori, S. G. Al-Ghamdi, S. Boulfrad, and M. Koç, "Life Cycle Assessment for Integration of Solid Oxide Fuel Cells into Gas Processing Operations," *Energies,* vol. 14, no. 15, 2021, doi: 10.3390/en14154668.
[33] H. Wang, P. Zhou, and Z. Wang, "Reviews on current carbon emission reduction technologies and projects and their feasibilities on ships," *Journal of Marine Science and Application,* vol. 16, no. 2, pp. 129-136, 2017, doi: 10.1007/s11804-017-1413-y.
[34] K. H. M. Al-Hamed and I. Dincer, "A comparative review of potential ammonia-based carbon capture systems," *J Environ Manage,* vol. 287, p. 112357, Jun 1 2021, doi: 10.1016/j.jenvman.2021.112357.
[35] X. Luo and M. Wang, "Study of solvent-based carbon capture for cargo ships through process modelling and simulation," *ApEn,* vol. 195, pp. 402-413, 2017, doi: 10.1016/j.apenergy.2017.03.027.
[36] L. Giordano, D. Roizard, and E. Favre, "Life cycle assessment of post-combustion CO 2 capture: A comparison between membrane separation and chemical absorption processes," *International Journal of Greenhouse Gas Control,* vol. 68, pp. 146-163, 2018, doi: 10.1016/j.ijggc.2017.11.008.
[37] R. Kleijn, E. van der Voet, G. J. Kramer, L. van Oers, and C. van der Giesen, "Metal requirements of low-carbon power generation," *Energy,* vol. 36, no. 9, pp. 5640-5648, 2011, doi: 10.1016/j.energy.2011.07.003.
[38] M. Wentker, M. Greenwood, and J. Leker, "A Bottom-Up Approach to Lithium-Ion Battery Cost Modeling with a Focus on Cathode Active Materials," *Energies,* vol. 12, no. 3, 2019, doi: 10.3390/en12030504.
[39] M. Chordia, A. Nordelöf, and L. A.-W. Ellingsen, "Environmental life cycle implications of upscaling lithium-ion battery production," *The International Journal of Life Cycle Assessment,* 2021, doi: 10.1007/s11367-021-01976-0.
[40] *Siemens Simotics*, 1RN7716-6NA10-0CG0-Z, 2021.
[41] *Siemens: Sigentics M generators*, PDLD-B10133-00-7600, 2018. [Online]. Available: siemens.com/sigentics-m.
[42] GREET Database.
[43] B. Greening and A. Azapagic, "Domestic heat pumps: Life cycle environmental impacts and potential implications for the UK," *Energy,* vol. 39, no. 1, pp. 205-217, 2012, doi: 10.1016/j.energy.2012.01.028.
[44] International Maritime Organization, "Reduction of GHG emissions from Ships: Fourth IMO GHG Study 2020," International Maritime Organization, London, 2021.
[45] K. Andersson and H. Winnes, "Environmental Trade-Offs in Nitrogen Oxide Removal from Ship Engine Exhausts," *Proc. Inst. Mech. Eng., Part M: J. Eng. Marit. Environ.,* vol. 225, no. 1, pp. 33-42, 2011, doi: 10.1243/14750902jeme223.
[46] E. Fridell, H. Salberg, and K. Salo, "Measurements of Emissions to Air from a Marine Engine Fueled by Methanol," *Journal of Marine Science and Application,* vol. 20, no. 1, pp. 138-143, 2020, doi: 10.1007/s11804-020-00150-6.

[47] S. Brynolf, M. Magnusson, E. Fridell, and K. Andersson, "Compliance possibilities for the future ECA regulations through the use of abatement technologies or change of fuels," *Transportation Research Part D: Transport and Environment,* vol. 28, pp. 6-18, 2014, doi: 10.1016/j.trd.2013.12.001.

[48] Wärtsilä Corporation. "World's first full scale ammonia engine test - an important step towards carbon free shipping." https://www.wartsila.com/media/news/30-06-2020-world-s-first-full-scale-ammonia-engine-test---an-important-step-towards-carbon-free-shipping-2737809 (accessed 05 May, 2021).

[49] MAN Energy Solutions. "MAN B&W two-stroke engine operating on ammonia." MAN Energy Solutions,. https://www.man-es.com/docs/default-source/marine/tools/man-b-w-two-stroke-engine-operating-on-ammonia.pdf?sfvrsn=544dc811_16 (accessed 05 May, 2021).

[50] S. Frigo, R. Gentili, and N. Doveri, "Ammonia Plus Hydrogen as Fuel in a S.I. Engine: Experimental Results," presented at the SAE Technical Paper Series, 2012.

[51] S. Grzesiak, "Alternative Propulsion Plants for Modern LNG Carriers," *New Trends in Production Engineering,* vol. 1, no. 1, pp. 399-407, 2018, doi: 10.2478/ntpe-2018-0050.

[52] *Uniturbo® 34FY – Centrifugal Compressor for large scale refrigeration plants and heat pumps*, G007-05, 2020.

[53] "London Metal Exchange." www.lme.com (accessed 15 January, 2022).

[54] "Scrap Monster." https://www.scrapmonster.com/ (accessed 22 January, 2022).

[55] "i scrap app." https://iscrapapp.com/ (accessed 22 January, 2022).

[56] K. Holmgren *et al.*, "KNOGA. Fossilfri framdrift för tunga långväga transporter på väg – Kostnadsfördelning och risker för olika aktörer," FDOS 13:2021, 2021. [Online]. Available: https://f3centre.se/sv/samverkansprogram/

[57] M. Taljegard, S. Brynolf, M. Grahn, K. Andersson, and H. Johnson, "Cost-Effective Choices of Marine Fuels in a Carbon-Constrained World: Results from a Global Energy Model," *Environ. Sci. Technol.,* vol. 48, no. 21, pp. 12986-12993, 2014/11/04 2014, doi: 10.1021/es5018575.
